# DNA extracted from boiled archival fish bones yields high‐quality whole‐genome sequencing data

**DOI:** 10.1111/jfb.70359

**Published:** 2026-04-09

**Authors:** Jingyao Niu, Anti Vasemägi, María‐Eugenia López, Lilian Pukk, Magnus Huss, Anna Gårdmark

**Affiliations:** ^1^ Swedish University of Agricultural Sciences Department of Aquatic Resources Uppsala Sweden; ^2^ Department of Population Analysis and Monitoring Swedish Museum of Natural History Stockholm Sweden; ^3^ Department of Aquatic Resources, Institute of Freshwater Research Swedish University of Agricultural Sciences Uppsala Sweden; ^4^ Chair of Aquaculture, Institute of Veterinary Medicine and Animal Sciences Estonian University of Life Sciences Tartu Estonia

**Keywords:** archival samples, DNA extraction, environmental monitoring, Eurasian perch *Perca fluviatilis*, fisheries institutions, fisheries management, operculum bones

## Abstract

Archival samples provide a unique source of organismal DNA, offering the potential to extend the temporal scale of genetic studies by decades to centuries. Fish hard structures, such as otoliths and scales, serve as records for fish collected during fisheries monitoring across a large spatiotemporal scale. Operculum bones are a type of fish hard structure that, although less commonly collected, have been utilised in genetic studies. However, the potential of archived operculum bones to provide high‐quality whole‐genome sequence information has not been thoroughly evaluated. Here, we applied a commercially available extraction protocol, with minor adjustments, to isolate genomic DNA from operculum bones of Eurasian perch collected up to 47 years ago, followed by standard next generation short‐read whole‐genome sequencing. By comparing the metrics of DNA and whole‐genome sequencing data of bones with contemporary muscle samples, we demonstrate that the application of a simple extraction protocol on boiled archival fish operculum bones yields high‐quality genomic information that is comparable to fresh tissue, highlighting the role of archival operculum bones as an overlooked repository of valuable genomic information.

## 1 INTRODUCTION

DNA analyses from historical samples provide opportunities to elucidate how past events or environmental conditions have shaped the demographic history of populations and species, track evolutionary changes over time and directly measure the rate of molecular evolution. Since the 1980s, researchers have studied ancient DNA (aDNA) stored in archaeological remains of humans, domestic animals and extinct megafauna (Hofreiter et al., 2015; Pääbo, 1993). In aquatic organisms, genetic data has been successfully sequenced from DNA isolated from fish bones stemming from the late Holocene (Ferrari et al., 2021; Martínez‐García et al., 2022). Skeletal remains may actually surpass soft tissue as a repository for DNA, potentially due to DNA's affinity to bind to bone minerals (Latham & Miller, 2018). However, analyses of aDNA have proven challenging, both in terms of acquiring biological material in archaeological excavations (Rizzi et al., 2012) and requirements for significant time, effort and expense because of the need for rigorous decontamination and quality control processes (Gilbert et al., 2006). Even when such decontamination regimes and authenticity protocols are followed, contaminants are frequently observed in the data (Kolman & Tuross, 2000). Furthermore, the opportunistic nature of most archaeological material often limits their temporal resolution, complicating efforts to associate genetic finding with climate‐induced temperature changes, and other environmental changes and anthropogenic stressors (Atmore et al., 2023; Ferrari et al., 2021).

**FIGURE 1:**
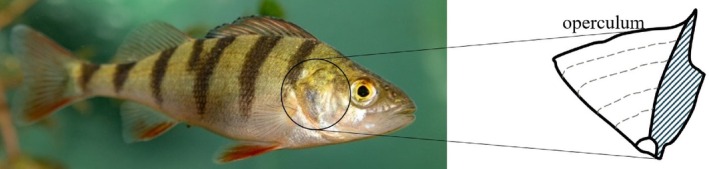
Schematic illustration of an operculum bone, which is part of the gill cover (here shown in a small Eurasian perch) and was used in DNA extraction. The dashed lines represent growth rings that signal the age of the fish. Material used in DNA extraction was primarily taken from the striped area to not disrupt further age reading from the operculum bone. Adjusted photograph of a Eurasian perch from Mark Harris.

Here, we focus on archival samples, another category of historical samples from which a significantly larger number of specimens have been regularly and consistently collected over the past 200 years (Wandeler et al., 2007). Thanks to the extensive efforts of fisheries institutes worldwide and their routine monitoring practices, there is a substantial collection of fish bony archival samples, including otoliths, scales, operculum bones, vertebrae, jaws and other parts of the bony endoskeleton (Campana & Thorrold, 2001; Nielsen & Hansen, 2008; Tzadik et al., 2017). A significant portion of these bony samples was collected for determining fish age and growth because they display visible annuli reflecting fish growth (Lai et al., 1995; Maunder & Punt, 2013). While age determination is typically performed using otoliths and scales, the operculum bone, located on the surface of the fish head as part of the gill cover (see Figure 1 for an example in Eurasian perch), serves as a better alternative for age determination (Le Cren, 1947; McConell, 1951) and morphological or chemical analyses in some fish species because of its flat shape and relatively large size (Huycke et al., 2012; Kusznierz et al., 2023; Tarasco et al., 2017). As a result, large collections of operculum bones exist for many species (Khan & Khan, 2009; Ma et al., 2011; Perry & Casselman, 2012; Thoresson, 1996).

Often stored in museums and research institutes, archives of fish bony samples constitute unparalleled spatiotemporal records of fish species and populations in the wild (Campana & Thorrold, 2001; Nielsen & Hansen, 2008). Since the late 1990s, the utility of archival samples as genetic repositories has primarily been for investigating the temporal stability of population structure, genetic diversity and effective population size (Manuzzi et al., 2022; Swatdipong et al., 2010). Many studies have since identified genetic consequences from fishing on wild fish populations (Cuveliers et al., 2009; Hutchinson et al., 2003; Miettinen et al., 2024; Price et al., 2019; Pukk et al., 2013). Other concurrent stressors in the environment, such as climate change, lack of food availability, parasitic load and disappearing habitat connectivity, have also been associated with evolutionary changes in fish through archival genetics (Björklund et al., 2015; Bonanomi et al., 2015; Czorlich et al., 2022; McDermid et al., 2014; Therkildsen et al., 2019). By analysing the genetic information collected from native, introduced and farmed populations, the genetic impact of historical stocking has been revealed (Finnegan & Stevens, 2008). Future stocking practice and spatial management units can be better informed and defined accordingly (Ciborowski et al., 2007; Östergren et al., 2021). Archival fish bony samples have demonstrated the ability to expand the time and space dimensions of evolutionary studies (Bi et al., 2013; Wandeler et al., 2007).

However, the majority of the molecular analyses performed using fish bony samples so far have been limited to a few mitochondrial DNA loci (Ciborowski et al., 2007) or microsatellite loci (Cuveliers et al., 2009; Price et al., 2019; Pukk et al., 2013). There is a growing number of studies utilising single nucleotide polymorphism (SNP) genotyping through reduced representation methods, such as restriction‐site associated DNA sequencing (RADseq, Jacobsen et al., 2017), SNP chips (Bonanomi et al., 2015; Johnston et al., 2013) or genotyping‐in‐thousands by sequencing (GT‐seq, Manuzzi et al., 2022; Setzke et al., 2021). To the best of our knowledge, only studies that have generated whole‐genome sequencing (WGS) data using archival bony samples from fish, to enable more robust evolutionary inferences and analyses of genomic variants beyond SNPs, have obtained DNA from fish scales and otoliths (Caccavo et al., 2024; Pinsky et al., 2021). Specifically, until now, no study has been done on archival operculum bones to test if such cellular bone tissue can yield DNA of enough quality to support subsequent WGS. Operculum bones may be particularly challenging for extraction of high‐quality DNA as they—in contrast to otoliths and scales—are commonly treated with boiling water to remove attached skin and tissue to clean the bone and increase growth ring readability.

In this study, we tested the possibility of generating WGS data by applying a commercially available DNA isolation kit, originally designed for blood and tissue samples, with only minor adjustments on archival operculum bones. Through a comparative analysis of DNA and sequencing quality from bones dated to the 1980s and 2000s, alongside fresh samples, we demonstrated that even boiled archival bones can effectively provide significant quantities of fish endogenous DNA in a manner that is both time‐efficient and cost‐effective, adequate for generating high‐quality WGS data.

## 2 MATERIALS AND METHODS

### 2.1 The boiled operculum bones

Like many institutes working with fish and fisheries, the Department of Aquatic Resources at the Swedish University of Agricultural Sciences (SLU Aqua) holds archives of dried fish bony tissues, some of which date back to more than 100 years ago. Among these archives, there is one for operculum bones from Eurasian perch (*Perca fluviatilis*) that has been maintained since 1970. The archive was established as an outcome of a coastal fish monitoring program that uses gillnets and fyke nets to track fish populations and environmental changes over time (Adill et al., 2013; Sandström et al., 1995; Thoresson, 1996). The operculum bones were sampled and prepared as follows: (1) removal of the left operculum of each individual; (2) each operculum bone was placed in a separate plastic chamber; (3) boiling water was poured over each chamber to cover the bone; (4) once the boiling water had cooled down, attached soft tissue on the bone was removed by brushing and rinsing with tap water; and (5) the bone was left to dry in a small paper envelope before being translocated into an archive room. Thus, the archival bones were treated with boiling water to deliberately remove attached tissues rich in DNA, which is far from optimal for isolating and preserving high‐quality DNA.

To evaluate (1) whether it is possible to extract DNA of enough quantity and quality from boiled archival operculum bones for WGS and (2) whether the storage time of bones affects the quality of DNA and WGS data, we selected a subset of 173 bone samples from this archive (Table S1). The subset contained bones collected from 1977 to 1978 (hereafter 1980s bones) and 2001–2002 (2000s bones) from two perch populations on the Baltic Sea coast of Sweden. The bones were selected by size to achieve sufficient sample weight (>10 mg). The body length of fish from which the operculum bones were sampled ranged from 166 to 340 mm (Figure S1).

### 2.2 Contemporary samples

To further evaluate how the type of tissue (bone/muscle), boiling and storage time of the bones affect quality of DNA and WGS data, we also used 105 contemporary perch muscle tissue samples collected from gillnet‐fishing in 2021 and 2022 from the same two populations. Approximately 1–3 cm^3^ of muscle was taken along the dorsal fin after removing the skin, using a clean scalpel and tweezers. The muscle samples were stored in 5 mL Eppendorf tubes filled with 95% ethanol and kept at 4°C. The tools and cutting board were cleaned and sterilised using 70% ethanol in between processing each individual. For simplicity, we refer to these muscle samples as the 2020s muscles.

### 2.3 DNA extraction

All DNA extractions were conducted in a designated DNA laboratory with as many steps as possible done under a stamina flow hood to minimise potential cross‐contamination and a strict unidirectional movement to avoid contact between pre‐ and post‐PCR samples. Disposable equipment, such as gloves, were changed after every potential contact with any sample (including touching the opening of any tubes). Non‐disposable equipment such as tweezers, bead‐beating beads and work surfaces were cleaned with bleach (0.5% sodium hypochlorite solution), Milli‐Q water and 70% ethanol. One extraction negative was included for every 24 samples.

For the 173 bone samples, the operculum bones were broken into smaller pieces to fit into 2 mL polypropylene screw cap micro tubes. To avoid contamination that can be introduced in additional handling of the bone pieces, we did not measure the bone piece weight used for each sample. Metal tweezers were used to break the bone if it was too hard to break by hand. One piece of every sample was re‐archived into the original paper envelope for potential future studies. Five 3 mm tungsten carbide beads (Qiagen) were added to the tube and the sample was homogenised by a PowerLyzer 24 Homogeniser (Qiagen) for 10 cycles of 30 s at speed of ~5000 m/s plus 30 s dwell time to avoid overheating. This resulted in a total of 10 min of homogenising at the maximum speed at which the micro tubes did not start to crack. The homogenising pulverised the bone samples. Subsequently, samples were centrifuged for 5 min at high speed (11,000 g) to settle the bone powder.

**FIGURE 2:**
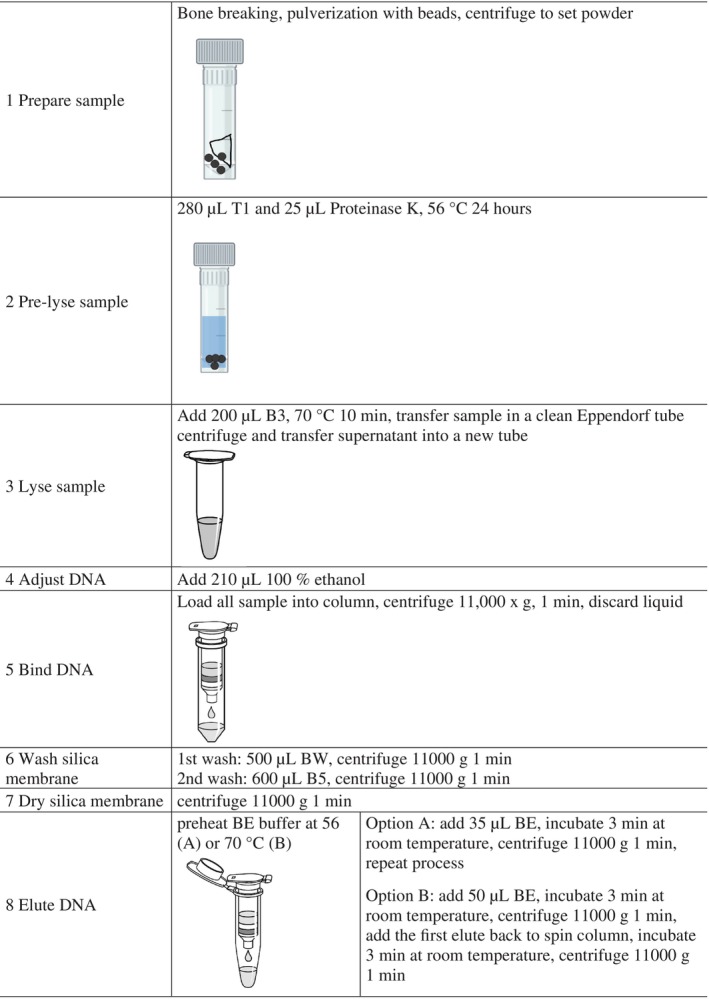
Graphic summary of the main steps of the DNA extraction protocol from fish operculum bones. Adapted from the Macherey‐Nagel NucleoSpin Tissue kit manual.

For subsequent extraction steps, we chose the Macherey‐Nagel NucleoSpin Tissue kit (#740952.250) and followed a slightly modified version of their standard protocol. The DNA extraction steps are summarised in Figure 2.

For bone powder digestion, we increased the amount of reagents used at multiple steps. First, all samples were digested in 280 μL of Buffer T1 and 25 μL of Proteinase K at 56°C for 24 h. Tubes were vortexed twice during the process with even time intervals. Digestion was finished with the addition of 200 μL of B3 Buffer and then kept at 70°C for 10 min. To remove the tungsten carbide beads from each sample, we transferred the supernatant from the micro tube to a sterile 1.5 mL Eppendorf tube. We centrifuged the 1.5 mL tube for 5 min at high speed (11,000 g) and transferred the supernatant again to a new 1.5 mL Eppendorf tube to remove any indigestible bone pieces from the sample before we proceeded with the DNA extraction.

Thereafter, we added 210 μL of 100% ethanol to the sample and vortexed the mixture vigorously. The mixture was then loaded into the NucleoSpin Tissue Column. DNA was bound to the silica membrane inside the NucleoSpin Tissue Column during the process of the mixture being washed through during centrifuging (11,000 g). We washed impurities off the silica membrane using 500 μL of Buffer BW and 600 μL of Buffer B5 following the standard protocol.

Finally, to collect as much DNA bound to the silica membrane as possible, we experimented with two alternative elution steps (A and B) on different bone samples to maximise the elution efficiency (Table S1). In total, 91 bone samples were treated with elution option A and 82 bone samples with elution option B (see Figure S1 for details). Option A used elution buffer pre‐heated to 56°C, with addition of 35 μL 56°C elution buffer to the silica membrane and incubation at room temperature for 3 min, following centrifugation (11,000 g, 1 min) and a second addition of 35 μL 56°C elution buffer following the same incubation and centrifugation steps. Option B used elution buffer pre‐heated to 70°C, with addition of 50 μL 70°C elution buffer, incubation at room temperature for 3 min and centrifugation at 11000 g for 1 min and a repetition of this last step using the first elute.

For the 105 muscle samples, we subsampled approximately 0.5 cm^3^ of muscle from each muscle sample to extract DNA following the standard protocol without modifications.

### 2.4 Validation of perch endogenous DNA

The DNA concentration of each sample was measured with a Qubit 4 Fluorometer (ThermoFisher Scientific). We used Qubit 1x dsDNA High Sensitivity (HS) and Broad Range (BR) assay kits to cover the 0.1–4000 ng range for both archival bone and contemporary muscle samples.

DNA fragment size distribution was assessed using electrophoresis run on an Agilent 2100 Bioanalyzer system using a DNA 7500 kit. We selected 12 samples (Tables S1 and S2) consisting of three individuals from the 1980s and 2000s samples from the two studied populations each to evaluate how storage time affects DNA fragmentation.

To validate the extraction of endogenous perch DNA from the bones, we performed end‐point PCR (on a BIOER thermal cycler) using the Qiagen Type‐it microsatellite PCR kit with two microsatellite primers designed for perch using a subset of all DNA samples that were processed before all other samples. The primers were Pflu4_5 (forward: 5′‐TTG ACA TAG CGG TCA AGT CTG T‐3′; reverse: 5′‐GAT TTG GAT GAC TTG CGT AGG‐3′; R. Gross, unpublished) and Pflu4_42 (forward: 5′‐CGG ACC AGG TTT CCT ACA GA‐3′; reverse: 5′‐TGA CTC CAT AAC CCT CCA CA‐3′; R. Gross, unpublished). The primers amplified microsatellite loci with size ranges of 115–147 bp (Pflu4_5) and 282–306 bp (Pflu4_42) which enabled us to evaluate the amplification of fragments with different sizes. Reaction components were added following the Type‐it Microsatellite PCR (Qiagen) protocol. The PCR reaction of total volume 15 μL contained 0.3 μL of each four primer of concentration 10 pmol/μL (final concentration in the reaction 0.2 μM), 7.5 μL of 2× Type‐it Multiplex PCR Master Mix (final concentration 1×), 1.3 μL of RNase‐free water and 5 μL of extracted DNA of concentration <10 ng/μL. The cycling protocol was as follows: 95°C for 5 min, 35 cycles at 95°C for 30 s, 61°C for 90 s and 72°C for 30 s, and a final extension at 60°C for 30 min. PCR products were run on 1% agarose gel stained with ethidium bromide and visualised under a UV light (Bio‐Rad GelDoc Go Gel Imaging System).

### 2.5 Library construction and sequencing

We chose 222 samples (148 bones and 74 muscles; see Table S3) of various DNA concentration (bones 0.6–48.5 ng/μL, muscles 54–256 ng/μL) and submitted them to Beijing Genomics Institute (BGI) for library preparation and short‐read WGS using the DNBSEQ platform: BGI‐SEQ 500 (Goodwin et al., 2016; Mak et al., 2017; Zhu et al., 2018). To construct the sequencing libraries of bone DNA samples, the KAPA HyperPrep Kit (Roche) was used as it is designed for highly fragmented DNA in low quantities. To minimise cost, the regular DNA Library Prep Kit (Yeasen) was used for the muscle DNA samples as there was no reason to motivate us to process them out of the ordinary (compared to the bone samples). We aimed for 10× sequencing depth across the genome with read length of 100 bp on pair end mode. No customisations were applied during these steps.

### 2.6 Bioinformatics

Read quality was assessed using FastQC version 0.11.9 (Andrews, 2019) and all sequences were trimmed with fastp v. 0.23.4 (Chen et al., 2018) applying the parameters:‐g‐w 12‐r‐W 5‐M 2‐trim_front1 9‐trim_front2 9‐trim_tail1 2‐trim_tail2 2‐l 60 due to the excess of polyG tails (De‐Kayne et al., 2021).

Filtered sequence reads of each individual were mapped to the Eurasian perch reference genome (NCBI: GCA_010015445.1) using bowtie2 (Langmead & Salzberg, 2012) and SAMtools v. 1.16 (Li et al., 2009). We applied default parameters, with the exception of the modified score minimum threshold (‐score‐min L, −0.3, −0.3) and the maximum fragment length for valid paired‐end alignments (−X 700). The proportions of mapped reads, coverage and depth were estimated using SAMtools v. 1.16 command flagstat and coverage.

Duplicated reads were identified and marked using Picard v. 2.27.5 MarkDuplicates (Broad Institure, 2022). Post‐mortem DNA damage, such as nucleotides substitution due to deamination (commonly found in aDNA samples; Ginolhac et al., 2011; Sawyer et al., 2012), was assessed using a subset of 36 randomly selected samples (12 1980s bones, 12 2000s bones and 12 2020s muscles) by Mapdamage2.0 (Jónsson et al., 2013) using default parameters. One of the degradation processes that DNA undergoes over time is cytosine deamination (Briggs et al., 2009), resulting in C to U changes at regular cytosines and C to T changes at 5‐methylated cytosines (Briggs et al., 2007). As cytosine deamination mostly occurs at the ends of ancient DNA fragments (Briggs et al., 2007), we quantified the level of DNA degradation by estimating the percentage of cytosine deamination within 25 bp from the forward read ends. Although the age of the bones was relatively young to be categorised as ancient, they were treated with boiling water that is known to degrade DNA.

We carried out Genome Analysis Toolkit (GATK) best practice pipeline v. 4.3.0.0 (Auwera and van der Auwera & O'Connor, 2020) to call variants for sequencing data. The HaplotypeCaller subroutine from GATK was applied to the BAM files to generate single‐sample GVCF files using the following parameters: ERC GVCF–minimum‐mapping‐quality 20‐mbq 13–G AS_StandardAnnotation. The CombineGVCF tool was then utilised to combine the individuals GVCF files into a single VCF file. Finally, consensus genotypes were called using the GenotypeGVCFs tool.

Initially, 12,775,282 SNPs were called using GATK Best Practices pipeline. Subsequently, we employed filtering and retained variants that met the following criteria using vcftools/0.1.16: (i) mean sequencing depth between 8 and 36 (−min‐meanDP 8–max‐meanDP 36), following (Ozerov et al., 2022); (ii) the consensus quality score of ≥30 (−minQ 30); (iii) minor allele frequency of ≥0.05 (MAF, −maf 0.05); (iv) missing in at most 5% of all individuals (−max‐missing 0.95); (v) biallelic sites and not indels (−max‐alleles 2–min‐alleles 2–remove‐indels); and (vi) Hardy–Weinberg equilibrium threshold no smaller than 0.01 (−hwe 0.01).

**FIGURE 3:**
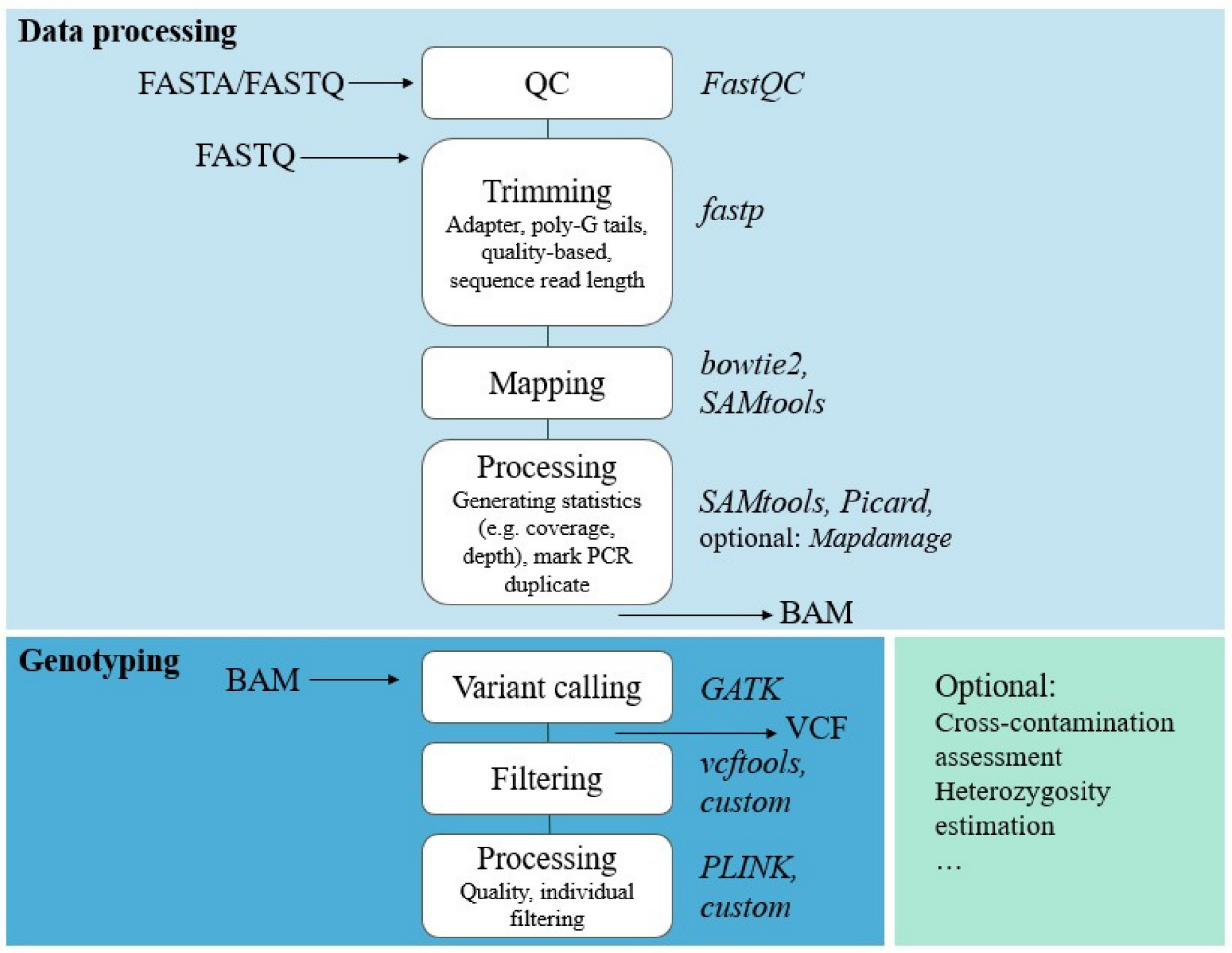
Overview of the bioinformatic pipeline from the raw data to sequencing statistics and processed files that are ready for subsequent analyses.

The treatments applied to the archive bones during sampling, for example bulk processing when removing operculum from the fish without following strict disinfection practices, can potentially increase the likelihood of cross‐sample contamination in DNA (when more than one individuals' DNA are unintentionally mixed). To evaluate this, we calculated observed heterozygosity (*H*_O_) using mitochondrial and nuclear SNPs (hereafter mtDNA *H*_O_ and nDNA *H*_O_) for each sample using PLINK v. 1.90b4.9–het (Chang et al., 2015) because abnormal/inaccurate estimates of *H*_O_ are signs of cross‐contamination. False heterozygotes are known to inflate measures of observed heterozygosity (Jun et al., 2012). Abnormality in *H*_O_ is evaluated among individuals. Since mtDNA is maternally‐inherited, each individual theoretically has only one haplotype in their mitogenome, and thus individual mtDNA *H*_O_ should be 0. When mtDNA *H*_O_ deviates from 0, it indicates potential mixing of mtDNA from other individuals. We evaluated whether an individual showed excess or deficiency of heterozygosity by visualising the distribution of individual *H*_O_ per population and time point. Because we have higher confidence in a lower contamination level for the contemporary samples, that is, the 2020s muscles, we used the *H*_O_ of them as a guide to signal the levels on both nDNA and mtDNA. The bioinformatics filtering steps are summarised in Figure 3.

### 2.7 Statistical analysis

All data processing and statistical analyses were conducted in R, v. 4.3.3 (R Core Team, 2025). Within bones, we used analysis of variance (ANOVA) to test if storage time and elution options affected their DNA yield and sequencing qualities. Among all samples, we tested if sequencing metrics differed between time points and tissue types (1980s bone, 2000s bone and 2020s muscle) using ANOVA. Data visualisation and processing were done using the packages within the tidyverse collection (Wickham et al., 2019).

## 3 RESULTS

### 3.1 DNA yield and fragmentation

**FIGURE 4:**
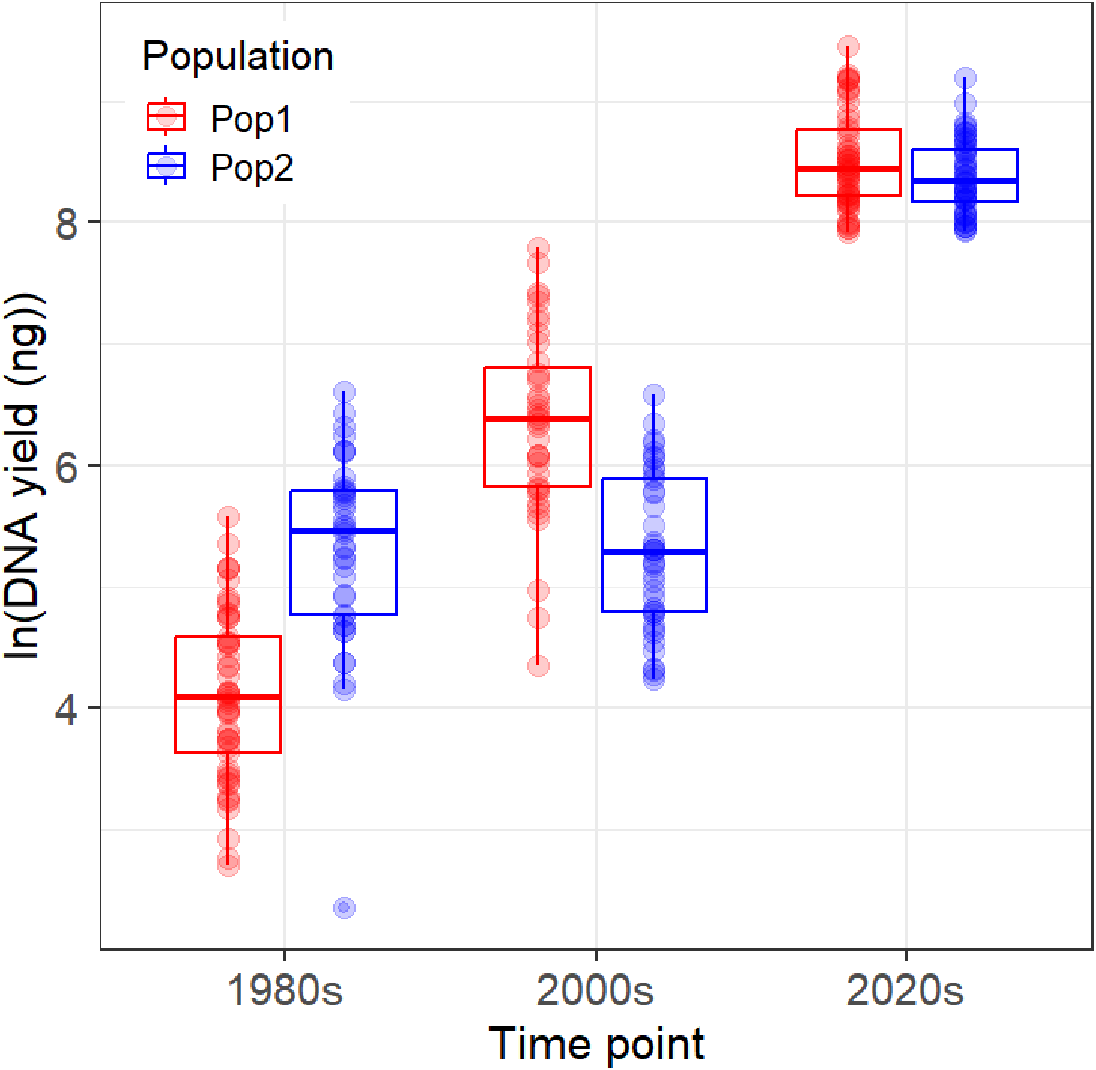
DNA total yield (in ng) shown after natural log transformed of the 1980s bone, 2000s bone and 2020s muscle samples from Population 1 (red) and Population 2 (blue). Each point represents an individual DNA sample.

Among all processed bones, the average DNA yield was 307.2 ng (10.6–2425.0 ng, eluded to either 50 or 70 μL; DNA concentration for 50 μL samples (82 samples): average 6.14, range 0.21–48.50 ng/μL; DNA concentration for 70 μL samples (91 samples): average 4.39, range 0.21–30.60 ng/μL), while the average DNA yield for muscles was 4972.0 ng (2740–12,800 ng; Figure 4, and Tables S1 and S4). Within bones, the average DNA yield was 153.8 ng for the 1980s bones and 485.5 ng for the 2000s bones. Elution option B using elution buffer pre‐heated to 70°C DNA yielded a higher amount of DNA for both the 1980s and 2000s bones than elution option A, likely due to more bone material being used relative to samples that underwent option A as DNA yield is positively correlated with fish body length (Figure S1). Whether there is a difference in elution efficiency between options A and B remains unclear. We recommend a more thorough experimental design if this is the goal of your research question. Within bones treated with the same elution option, the 2000s bones yielded a higher amount of DNA compared to the 1980s bones (ANOVA, elution option A: *F*(1, 89) = 12.63, *p* = 0.61e‐4; elution option B: *F*(1, 80) = 77.25, *p* = 2.28e‐13), suggesting a negative effect of storage time on DNA yield.

The results of the gel run for the PCR products of 12 bone samples (Figure S2) showed that the amplification of both microsatellite loci was successful for the majority of samples, indicating that the extracted DNA was indeed endogenous perch DNA, with fragments sizes of at least 300 bp. The bioanalyzer results (Figure S3) showed that most fragments in the 1980s bones were around 300 bp long, while the 2000s bone DNA samples had fragments from 300 bp to up to more than 10,000 bp, with a peak at around 7000 bp.

### 3.2 Sequencing, mapping and read quality

**FIGURE 5:**
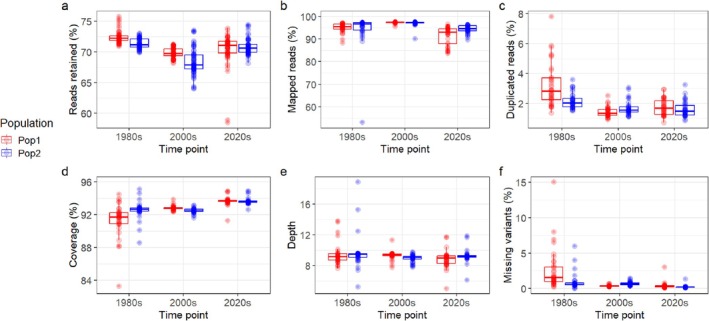
Sequence metrics and mapped reads statistics of the 1980s bone, 2000s bone and 2020s muscle samples from Population 1 (red) and Population 2 (blue): (a) the proportion of reads retained after trimming (1980s > 2020s > 2000s, *p* < 0.001); (b) the proportion of reads mapped to the reference genome (2000s > 1980s > 2020s, *p* < 0.001); (c) the percentage of duplicated reads (2000s < 2020s < 1980s, *p* < 0.001); (d) the percentage of the genome covered by mapped reads (2020s > 2000s > 1980s, *p* < 0.001); (e) the average depth (the number of times a specific base is covered by sequence reads,1980s > 2000s > 2020s, but only the difference between 1980s and 2020s is statistically significant: *p* = 0.019), and (f) the percentage of missing variants for each individual (1980s > 2000s > 2020s, *p* < 0.001). Each point represents the genomic data of one individual.

On average, 70% of reads across all samples were retained after trimming (Figure 5a). Rather unexpectedly, the 1980s bones had the highest proportion of reads retained (mean 71.85%, range 69.90–75.77%), while the 2000s bones had the lowest (mean 69.08%, range 63.99–73.52%; ANOVA, *F*_2,409_ = 97.76, *p* < 0.001). In general, all samples showed a high proportion of mapped reads (>95%), except a single outlier individual (< 60%) from the 1980s (Figure 5b). The 2000s bones showed the highest quality with respect to the proportion of mapped reads (ANOVA, *F*_2, 219_ = 24.66, *p* < 0.001). The level of duplicated reads was low overall (0.7–7.8%), with the 1980s bone samples having the highest proportion of duplicates among the three time points (ANOVA, *F*_2, 219_ = 45.83, *p* < 0.001; Figure 5c).

Overall, genome coverage was high (>90%) across all samples and time points (Figure 5d). Bones from the 1980s had the lowest coverage and most variation among samples, while the contemporary muscle samples showed the highest coverage (>92%, ANOVA, *F*_2, 219_ = 48.93, *p* < 0.001). Sequencing depth differed minimally between studied samples and time points (Figure 5e, ANOVA, *F*_2, 219_ = 3.07, *p* = 0.048, although highest for 1980s with an average of 9.5, coverage at only 2020s was significantly lower [*p* = 0.019] but with only a 0.47 difference, 0.36 higher than 2000s but not significant [*p* = 0.073]), with most samples exceeding 8‐fold read depth.

After the filtering, 908,800 SNPs were retained for the nuclear genome and ~300 SNPs for the mitogenome. The percentage of missing variants was low for both bone and muscle samples across all time periods, with mean missing percentage decreasing over time (ANOVA, *F*_2, 219_ = 24.22, *p* < 0.001; 1980s: 1.73 ± 5.27%, 2000s: 0.52 ± 0.06% and 2020s: 0.29 ± 0.13%) and the largest variance within the 1980s bones, with some individuals missing more than 5% (Figure 5f).

**FIGURE 6:**
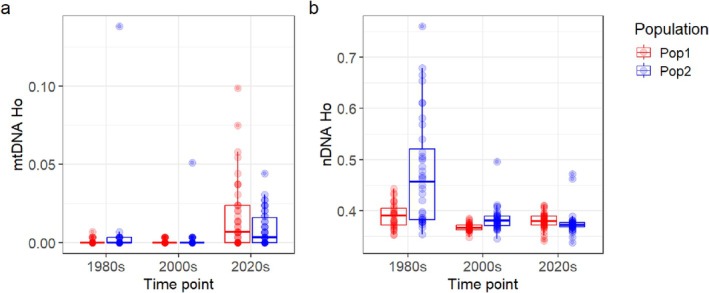
(a) Observed heterozygosity estimated using single nucleotide polymorphisms (SNPs) from mitochondrial DNA (mtDNA H_O_) and (b) observed heterozygosity estimated using nuclear SNPs (nDNA H_O_) for the 1980s bone, 2000s bone and 2020s muscle samples from Population 1 (red) and Population 2 (blue). Each point represents one individual.

Using mitochondrial information, a total of six samples exhibited mtDNA *H*_O_ levels above 0.05 (relatively high and appearing to be outliers compared to the rest of the samples), including one sample each from the 1980s and 2000s, and four samples from the 2020s, as shown in Figure 6a. Based on nuclear SNP data, a total of 11 individuals (eight samples from the 1980s, one from the 2000s and two from the 2020s) exhibited nDNA *H*_O_ > 0.45 (similarly, relatively high compared to the rest of the samples; Figure 6b). However, the excess heterozygosity patterns revealed based on mtDNA *H*_O_ (Figure 6a) and nDNA *H*_O_ (Figure 6b) did not match each other: samples with the highest mtDNA *H*_O_ values were collected in the 2020s, whereas samples with the highest nDNA *H*_O_ estimates were from the 1980s.

DNA post‐mortem damage, as indicated by estimated deamination levels at the forward ends of DNA strands, appeared to be low (<1%) across all samples (Figure S4). The 2000s bones and 2020s muscles were estimated to share similar levels of deamination while some of the 1980s bones displayed 2‐ to 4‐fold higher deamination levels, which were still too low to require further processing.

## 4 DISCUSSION

We tested the feasibility of extracting DNA from archival boiled fish operculum bones using a simple protocol. Not only did we succeed, we also compared the quality of subsequent WGS data from the boiled fish bones to contemporary muscle tissue. Here, we present our observations made in the process.

As expected, the bone samples yielded significantly less DNA than the muscle samples in terms of both quantity and concentration (Figure 4). However, since bones and muscles are two types of tissues of different properties, and we did not control for the amount of material used, the comparison on DNA yield was made between bones of different storage times. The negative effect of storage time (40+ and 20+ years) on DNA yield could be due to the ~20 additional years of DNA degradation when stored dry at room temperature (Nielsen & Hansen, 2008). In addition, DNA extracted from samples with a storage time of 40+ years had much shorter fragments than DNA from samples with storage time of 20+ years. Storage time itself likely affected both the level of DNA degradation and fragmentation.

To recover the maximal amount of DNA bound to the silica membrane, we experimented with two elution options and did not observe a clear difference in elution efficiency between the two options. Controlling the amount of bone material used in the experiment is needed to conclude anything concrete. Additionally, some DNA extraction protocols, such as the Monarch® Spin gDNA Extraction Kit (NEB #T3010S/L), recommend against preheating the elution buffer to temperatures above 60°C because hot elution may result in partial and irreversible denaturation of the eluted genomic DNA, depending on the salt conditions, but we did not observe any sequencing quality difference between samples that underwent the two elution options. Readers should exercise caution when determining the elution step and prioritise intended research goals whilst balancing the amount of additional time and labour required.

More importantly, based on the general patterns in sequencing metrics, the bones from the 1980s, 2000s and muscles from the 2020s all exhibited high sequence data quality (Figure 4). With the only exception of genome coverage (Figure 4d), the bones generally displayed equal or higher quality levels compared to the muscles, suggesting that neither the type of tissue, storage time nor boiling treatment had a major negative impact on the quality of the sequencing data. Notably, boiled archival operculum bones can yield WGS data of quality comparable to that of fresh muscle samples.

We evaluated the level of cross‐contamination by estimating the observed heterozygosity of each sample and were left with contradicting patterns based on mtDNA (Figure 5a) and nuclear DNA SNPs (Figure 5b). However, given that the number of nDNA SNPs is much larger than the number of mtDNA SNPs (900k and 300), it is likely that the observed heterozygosity estimates based on nuclear data provide more robust and reliable information about potential contamination than mtDNA *H*_*O*_. Thus, it is likely that the highest level of cross‐individual contamination was observed in samples from population 2 from the 1980s. This result also highlights batch‐effect in relation to cross‐contamination (during sampling) when studying archival samples. As the bones were originally collected for age and growth determination rather than DNA extraction (Thoresson, 1996), their sampling process lacked standard molecular analysis precautions and decontamination steps, such as changing gloves and sterilising boiling‐water chambers in between samples. In comparison, the muscle sampling process followed a more rigorous decontamination routine. We also observed a slightly increased level of DNA degradation in the 1980s bones compared to 2000s bones and 2020s muscles in terms of post‐mortem damage, but the overall very low level of deamination suggests that the additional 20 years of storage time (between 1980s and 2000s bones) and boiling treatment during bone sampling had little influence on sequencing quality. Nevertheless, we only detected a few low‐quality archival DNA samples using the mentioned criteria and those can be easily removed by filtering for downstream genomic analysis without costing too much time and money.

During recent years, first successful attempts have been published to obtain whole (as well as partial) genome sequencing data using excavated ancient fish bones, demonstrating that fish bones can be good sources of DNA (Atmore et al., 2023; Ferrari et al., 2021; Kirch et al., 2021; Laine et al., 2025). Studies extracting DNA using trace tissue attached to archival otolith and scale samples have also been reported (Caccavo et al., 2024; Pinsky et al., 2021). Similar extraction methods might be worth testing out on operculum bones too – ideally if the bones have not been treated with boiling – to further optimise the time, labour and expense consumption. Furthermore, recent studies have provided a much‐needed temporal perspective to investigate the association between concurrent environmental changes and evolutionary changes in wild fish populations (Caccavo et al., 2024; Pinsky et al., 2021). As a next step, we investigate the WGS data acquired from the two populations to identify potential selection signatures of rising water temperatures, aiming to provide evolutionary insights on how fish genomes evolve and change in the context of climate change.

We hope our demonstration of a simple and efficient DNA isolation protocol on bony samples will motivate further utilisation of archives in fisheries institutions and museums. Further optimisation of the extraction protocol from fish operculum bones would require additional investigation into factors such as bone thickness and size, which may influence DNA yield and quality. Understanding these relationships is important for developing effective protocols for genomic analyses using operculum bones. Accordingly, the field of fish genomics would benefit from a broader and more systematic evaluation of methods and protocols for DNA extraction from more types of archival bony material and the subsequent library preparation and sequencing practices. Our findings highlight that although the sampling (e.g. the boiling) and storage were suboptimal for molecular analyses, the utility of archival fish operculum bones for evolutionary studies is comparable to fresh muscle samples. Overall, the integration of genomic, archaeological and ecological approaches offers the potential to address a range of scientific and management questions that cannot be fully achieved by either discipline in isolation.

## AUTHOR CONTRIBUTIONS

J.N.: Conceptualization, data curation, formal analysis, investigation, methodology, visualisation, writing – original draft, writing – review and editing. A.V.: Conceptualization, investigation, methodology, funding acquisition, supervision, writing – original draft, writing – review and editing. M.E.L.: Data curation, formal analysis, investigation, methodology, supervision, writing – original draft, writing – review and editing. L.P.: Data curation, investigation, methodology, supervision, writing – review and editing. M.H.: Conceptualization, data curation, writing – review and editing. A.G.: Conceptualization, data curation, funding acquisition, supervision, writing – original draft, review and editing.

## Supporting information


**FIGURE S1.** Opaque grey circles in the left panel represent log‐transformed DNA yield from 1980s to 2000s bones extracted using elution options A and B, while the blue circles indicate the body length of the fish from which the bones were taken. The number of bone samples that underwent elution options A and B are listed on the top right panel. Statistically, elution strategy did not significantly influence DNA yield. DNA yield, however, correlated positively with fish body length (*p* = 0.00084, at a low coefficient 0.016). The summary stats are shown on the bottom right panel.
**FIGURE S2.** Multiplex PCR of *P. fluviatilis* microsatellite loci (Pflu4_5, 115–147 bp; Pflu4_42, 282–306 bp) using 12 bone samples visualised in 1% ethidium bromide stained agarose gel. The columns indicated as “Ladder” were Thermo Scientific GeneRuler 1 kb DNA ladders, used to indicate the fragment size (bp). The lowest band shown in all samples including the PCR negative control (PCRneg) was the amplification of the primers (~20 bp). Besides the primer band, all except bone sample 4 showed two bands, suggesting the amplification of both loci.
**FIGURE S3.** One example each of DNA fragment size distribution from the 1980s bones (left) and 2000s bones (right) analysed by the DNA 7500 kit for 2100 Bioanalyzer Systems. From low to high numbers along the *x* axis, each tick represents the 50, 100, 300, 500, 700, 1000, 1500, 2000, 3000, 5000, 7000 and 10,380 bp markers of the ladder. The 1980s bone shows only one bump just below 300 bp, indicating that most fragments were around 300 bp long. The 2000s bone shows a distribution curve that elevates from below 300 bp, slightly and slowly decreases after, and peaks around 7000 bp. This shows that a significant amount of fragments ranging from 300 to 10,000 bp were present in the 2000s sample.
**TABLE S2.** The key to the 12 bone samples presented in bioanalyzer.12sample.kit7500.pdf. They are also marked in Table S1.
**FIGURE S4.** Example of one 1980s bone (top) and one 2020s muscle (bottom) sample estimated cytosine to thymine mis‐incorporation (equals to G>A) frequency from the 25 first nucleotides of the forward and reverse DNA strands to signal DNA post‐mortem damage patterns.

**TABLE S1.** Information on operculum bones from which DNA was extracted and the fish from which the operculum bones were sampled. The letters ‘A’ and ‘B’ in column ‘Elution option’ correspond to the two alternatives within elution steps described in the main text. The 'Bioanalyzer sample ID' column correspondes to the 12 DNA samples in supplementary file 'bioanalyzer.12sample.kit7500.pdf'.

**TABLE S3.** Information on the 222 sequenced DNA samples, including their sequencing metrics.

**TABLE S4.** DNA concentration and total quantity from the 2020s muscle samples.

**DATA S1.** Supporting Information.
