# Supplementary material for: DNA extracted from boiled archival fish bones yields high‐quality whole‐genome sequencing data
Source: J Fish Biol. 2026 Apr 9;108(6):2137–47. doi: 10.1111/jfb.70359 (PMC13357346; doi:10.1111/jfb.70359)
Supplement: Supplementary file 1 — FIGURE S1. Opaque grey circles in the left panel represent log‐transformed DNA yield from 1980s to 2000s bones extracted using elution options A and B, while the blue circles indicate the body length of the fish from which the bones were taken. The number of bone samples that underwent elution options A and B are listed on the top right panel. Statistically, elution strategy did not significantly influence DNA yield. DNA yield, however, correlated positively with fish body length (p = 0.00084, at a low coefficient 0.016). The summary stats are shown on the bottom right panel. FIGURE S2. Multiplex PCR of P. fluviatilis microsatellite loci (Pflu4_5, 115–147 bp; Pflu4_42, 282–306 bp) using 12 bone samples visualised in 1% ethidium bromide stained agarose gel. The columns indicated as “Ladder” were Thermo Scientific GeneRuler 1 kb DNA ladders, used to indicate the fragment size (bp). The lowest band shown in all samples including the PCR negative control (PCRneg) was the amplification of the primers (~20 bp). Besides the primer band, all except bone sample 4 showed two bands, suggesting the amplification of both loci. FIGURE S3. One example each of DNA fragment size distribution from the 1980s bones (left) and 2000s bones (right) analysed by the DNA 7500 kit for 2100 Bioanalyzer Systems. From low to high numbers along the x axis, each tick represents the 50, 100, 300, 500, 700, 1000, 1500, 2000, 3000, 5000, 7000 and 10,380 bp markers of the ladder. The 1980s bone shows only one bump just below 300 bp, indicating that most fragments were around 300 bp long. The 2000s bone shows a distribution curve that elevates from below 300 bp, slightly and slowly decreases after, and peaks around 7000 bp. This shows that a significant amount of fragments ranging from 300 to 10,000 bp were present in the 2000s sample. TABLE S2. The key to the 12 bone samples presented in bioanalyzer.12sample.kit7500.pdf. They are also marked in Table S1. FIGURE S4. Example of one 1980s bone (top) and [file JFB-108-2137-s005.docx]

Supplementary materials of manuscript

DNA extracted from boiled archival fish bones yields high quality whole genome sequencing data

Niu J^1^, Vasemägi A^2,3^, López M-E^2^, Pukk L^3^, Huss M^1^ and Gårdmark A^1^

^1^ Swedish University of Agricultural Sciences, Department of Aquatic Resources, Box 7018, SE-750 07 Uppsala, Sweden

^2^ Swedish University of Agricultural Sciences, Department of Aquatic Resources, Institute of Freshwater Research, Stångholmsvägen 2, SE-178 93 Drottningholm, Sweden

^3^ Chair of Aquaculture, Institute of Veterinary Medicine and Animal Sciences, Estonian University of Life Sciences, Kreutzwaldi 46A, Tartu, Estonia

## Although we selected bones from fish of as similar sizes as possible, there is a wide range of fish sizes (Figure S1).


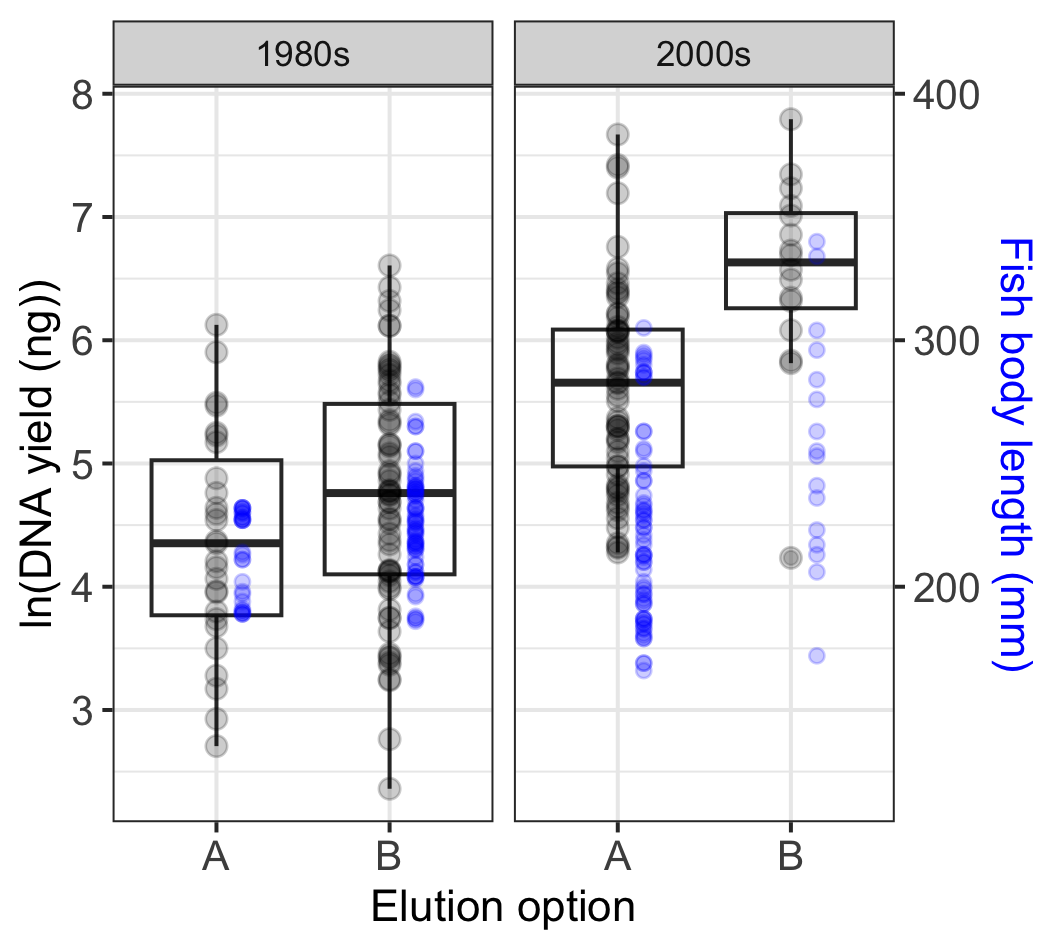

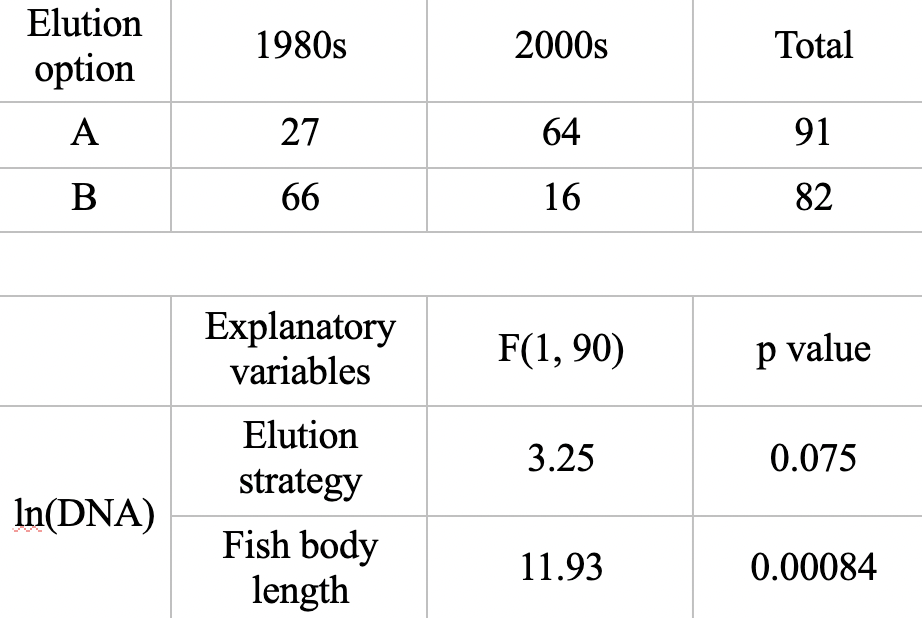


Figure S1. Opaque grey circles in the left panel represent log-transformed DNA yield from 1980s and 2000s bones extracted using elution options A and B, while the blue circles indicate the body length of the fish from which the bones were taken. The number of bone samples underwent elution option A and B are listed on the top right panel. Statistically, elution strategy did not significantly influence DNA yield. DNA yield, however, correlate positively with fish body length (p = 0.00084, at a low coefficient 0.016). The summary stats are shown on the bottom right panel.

##
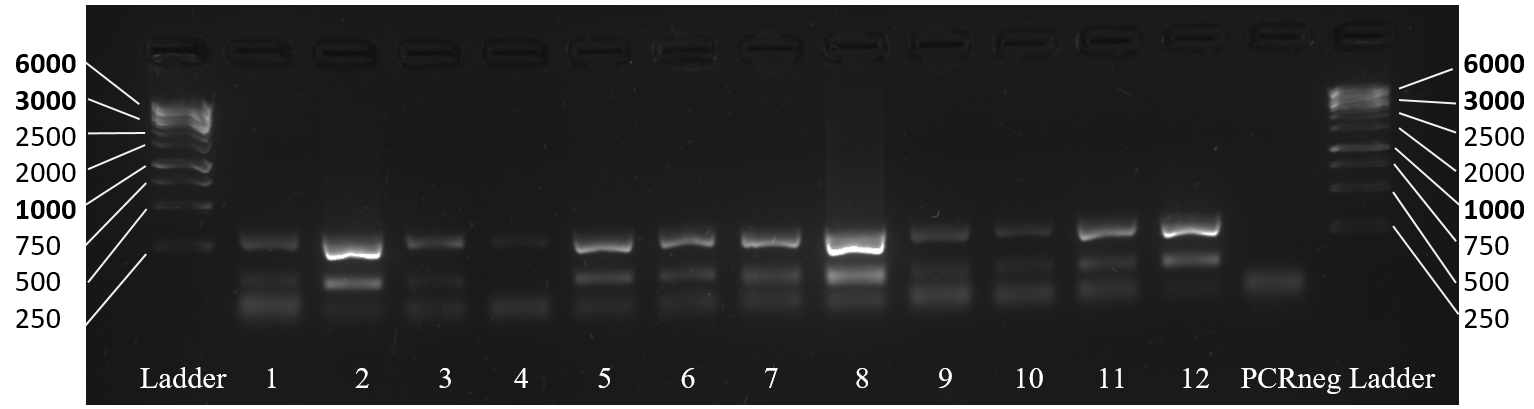


Figure S2. Multiplex PCR of P. fluviatilis microsatellite loci (Pflu4_5, 115-147 bp; Pflu4_42, 282-306 bp) using 12 bones samples visualized in 1% ethidium bromide stained agarose gel. The columns indicated as “Ladder” were Thermo Scientific GeneRuler 1 kb DNA Ladders, used to indicate the fragment size (bp). The lowest band shown in all samples including the PCR negative control (PCRneg) was the amplification of the primers (~20 bp). Besides the primer band, all except bone sample 4 showed two bands, suggesting the amplification of both loci.

We assessed the distribution of DNA fragment size of 12 DNA samples (six 1980s bones and six 2000s bones, Figure S2). We show one example each for the 1980s and 2000s in Figure S3. Bioanalyzer result of all 12 samples (Table S1) are shown in the supplementary file bioanalyzer.12sample.kit7500.pdf.


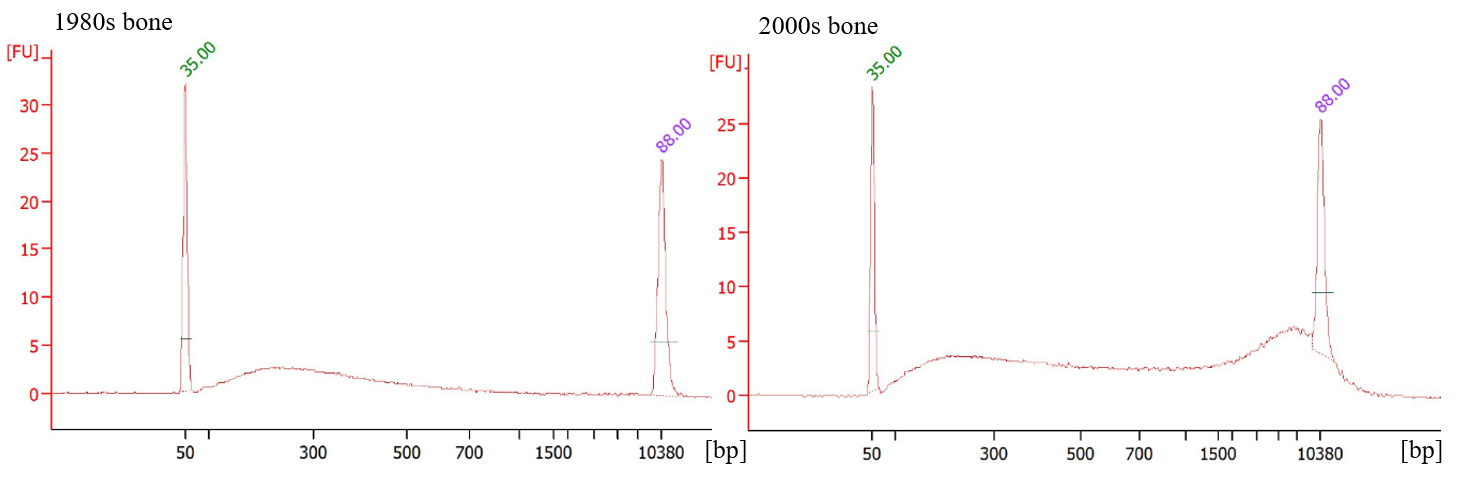


Figure S3. One example each of DNA fragment size distribution from the 1980s bones (left) and 2000s bones (right) analysed by the DNA 7500 kit for 2100 Bioanalyzer Systems. From low to high numbers along the x axis, each tick representing the 50, 100, 300, 500, 700, 1000, 1500, 2000, 3000, 5000, 7000 and 10380 bp marker of the ladder. The 1980s bone shows only one bump just below 300 bp, indicating that most fragments were around 300 bp long. The 2000s bone shows a distribution curve that elevates from below 300 bp, slightly and slowly decreases after, and peaks around 7000 bp. This shows that a significant amount of fragments ranging 300 - 10000 bp were present in the 2000s sample.

Table S2. The key to the 12 bone samples presented in bioanalyzer.12sample.kit7500.pdf. They were also marked in Table S1.

| Sample | Name | Time point |
| --- | --- | --- |
| 1 | Yao_sample_BT1 | 1980s |
| 2 | Yao_sample_BT2 | 1980s |
| 3 | Yao_sample_BT3 | 1980s |
| 4 | Yao_sample_BT4 | 2000s |
| 5 | Yao_sample_BT5 | 2000s |
| 6 | Yao_sample_BT6 | 2000s |
| 7 | Yao_sample_FM1 | 1980s |
| 8 | Yao_sample_FM2 | 1980s |
| 9 | Yao_sample_FM3 | 1980s |
| 10 | Yao_sample_FM6 | 2000s |
| 11 | Yao_sample_FM4 | 2000s |
| 12 | Yao_sample_FM5 | 2000s |


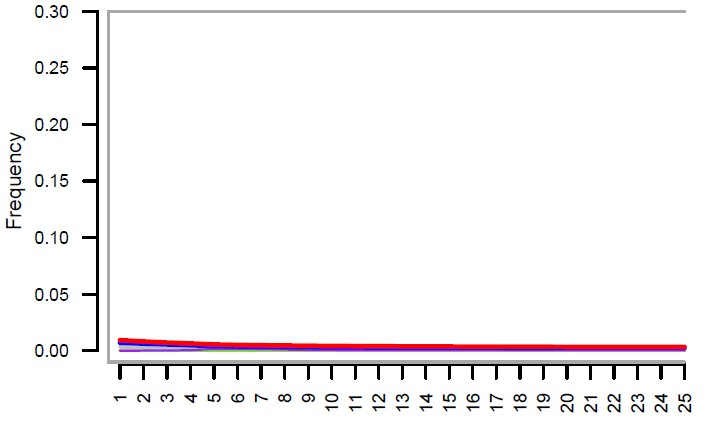


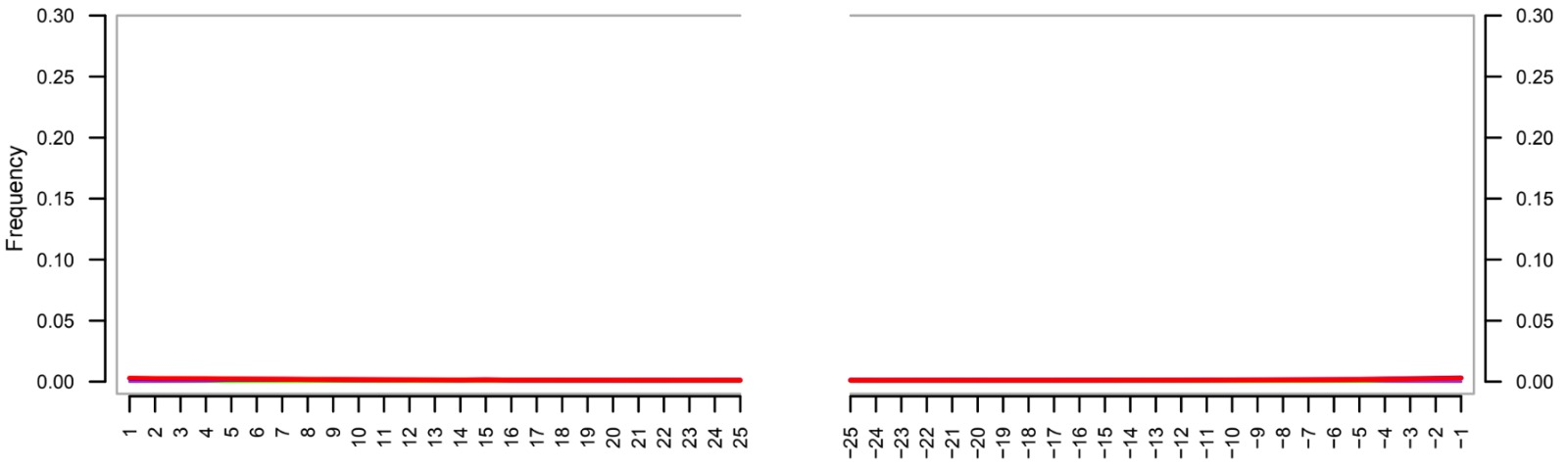


Figure S4. Example of one 1980s bone (top) and one 2020s muscle (bottom) sample estimated cytosine to thymine mis-incorporation (equals to G>A) frequency from the 25 first nucleotides of the forward and reverse DNA strands to signal DNA post-mortem damage patterns.
