# Supplementary material for: DNA extracted from boiled archival fish bones yields high‐quality whole‐genome sequencing data
Source: J Fish Biol. 2026 Apr 9;108(6):2137–47. doi: 10.1111/jfb.70359 (PMC13357346; doi:10.1111/jfb.70359)
Supplement: Supplementary file 5 — DATA S1. Supporting Information. [file JFB-108-2137-s003.pdf]

Assay Class: DNA 7500
Data Path: C:\...-28\2100 expert_DNA 7500_DE72901282_2024-06-28_14-30-41.xad

Created: 2024-06-28 14:30:41
Modified: 2024-06-28 15:14:09

Electrophoresis File Run Summary

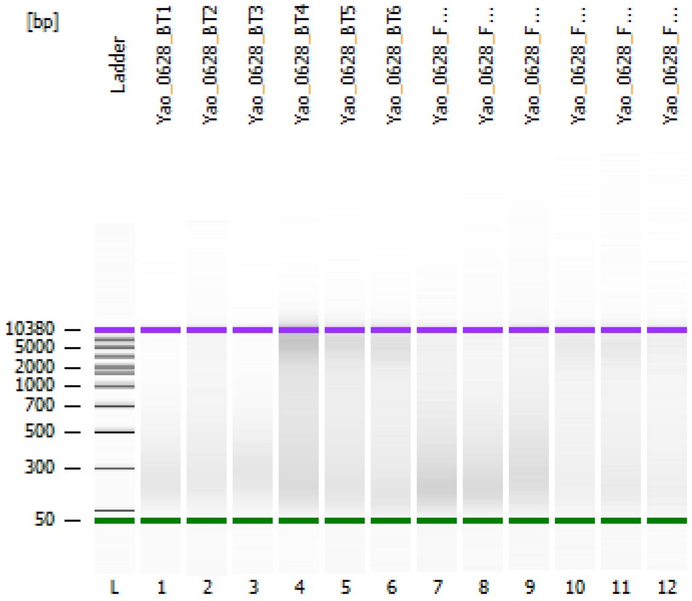

Instrument Information:

Instrument Name:	Bioanalyzer	Firmware:	C.01.069
Serial#:	DE72901282	Type:	G2939A

Assay Information:

Assay Origin Path:	C:\Program Files (x86)\Agilent\2100 bioanalyzer\2100 expert\Assays\dsDNA\DNA 7500 Series II.xsy
Assay Class:	DNA 7500
Version:	2.3
Assay Comments:	DNA Analysis 100 -7500 bp

© Copyright 2003-2009 Agilent Technologies, Inc.

Chip Information:

Chip Lot #:

Reagent Kit Lot #:

Chip Comments:

Yao_0628_BT1

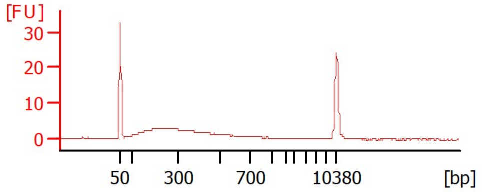

Yao_0628_BT2

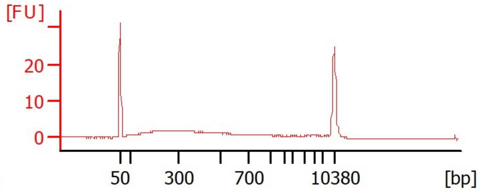

Yao_0628_BT3

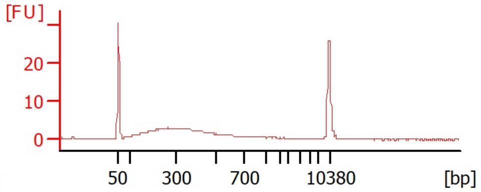

Yao_0628_BT4

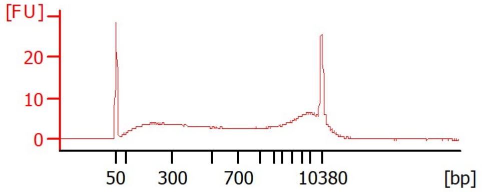

Yao_0628_BT5

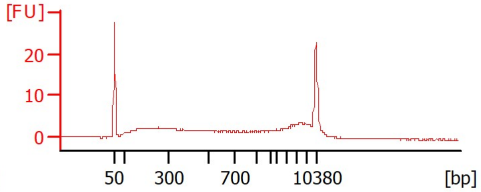

Yao_0628_BT6

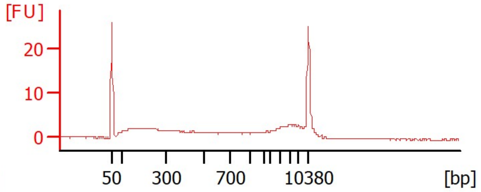

Yao_0628_FM1

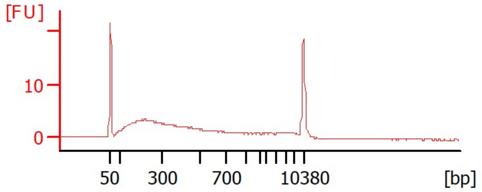

Yao_0628_FM2

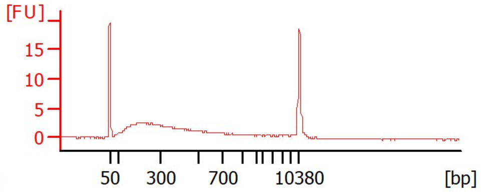

Yao_0628_FM3

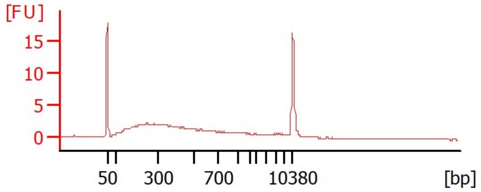

Yao_0628_FM4

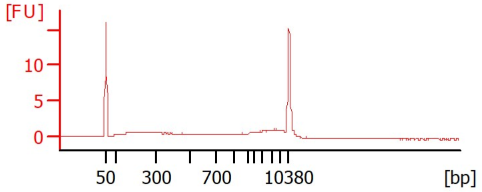

Yao_0628_FM5

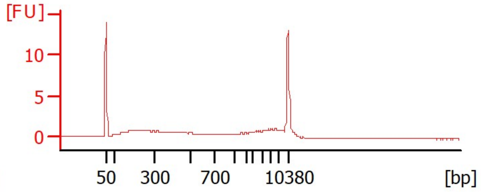

Yao_0628_FM6

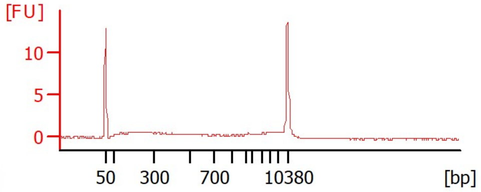

Assay Class: DNA 7500
Data Path: C:\...-28\2100 expert_DNA 7500_DE72901282_2024-06-28_14-30-41.xad
Created: 2024-06-28 14:30:41
Modified: 2024-06-28 15:14:09

Electrophoresis File Run Summary (Chip Summary)

Sample Name	Sample Comment	Rest. Digest	Status	Observation	Result Label	Result Color
Yao_0628_BT1		☐	✔			
Yao_0628_BT2		☐	✔			
Yao_0628_BT3		☐	✔			
Yao_0628_BT4		☐	✔			
Yao_0628_BT5		☐	✔			
Yao_0628_BT6		☐	✔			
Yao_0628_FM1		☐	✔			
Yao_0628_FM2		☐	✔			
Yao_0628_FM3		☐	✔			
Yao_0628_FM4		☐	✔			
Yao_0628_FM5		☐	✔			
Yao_0628_FM6		☐	✔			
Ladder		☐	✔			

Chip Lot # **Reagent Kit Lot #**

Chip Comments :

© Copyright 2003 - 2021 Agilent, Inc.

Assay Class: DNA 7500 Created: 2024-06-28 14:30:41
Data Path: C:\...-28\2100 expert_DNA 7500_DE72901282_2024-06-28_14-30-41.xad Modified: 2024-06-28 15:14:09

Electrophoresis Assay Details

General Analysis Settings

Number of Available Sample and Ladder Wells (Max.) : 13
Minimum Visible Range [s] : 20
Maximum Visible Range [s] : 94
Start Analysis Time Range [s] : 20
End Analysis Time Range [s] : 93,95
Ladder Concentration [ng/µl] : 40
Uses Standard Area for Ladder Fragments
Lower Marker Concentration [ng/µl] : 8,3
Upper Marker Concentration [ng/µl] : 4,2
Used Upper Marker for Quantitation
Standard Curve Fit is Point to Point
Show Data Aligned to Lower and Upper Marker

Integrator Settings

Integration Start Time [s] : 20
Integration End Time [s] : 93,95
Slope Threshold : 0,8
Height Threshold [FU] : 5
Area Threshold : 0,1
Width Threshold [s] : 0,5
Baseline Plateau [s] : 0,5

Filter Settings

Filter Width [s] : 0,5
Polynomial Order : 4

Ladder

Ladder Peak	Size	Area
1	50	120
2	100	47
3	300	63
4	500	81
5	700	85
6	1000	93
7	1500	101
8	2000	101
9	3000	106
10	5000	108
11	7000	109
12	10380	107

 © Copyright 2003 - 2021 Agilent, Inc.

Assay Class: DNA 7500 Created: 2024-06-28 14:30:41
Data Path: C:\...-28\2100 expert_DNA 7500_DE72901282_2024-06-28_14-30-41.xad Modified: 2024-06-28 15:14:09

Electropherogram Summary

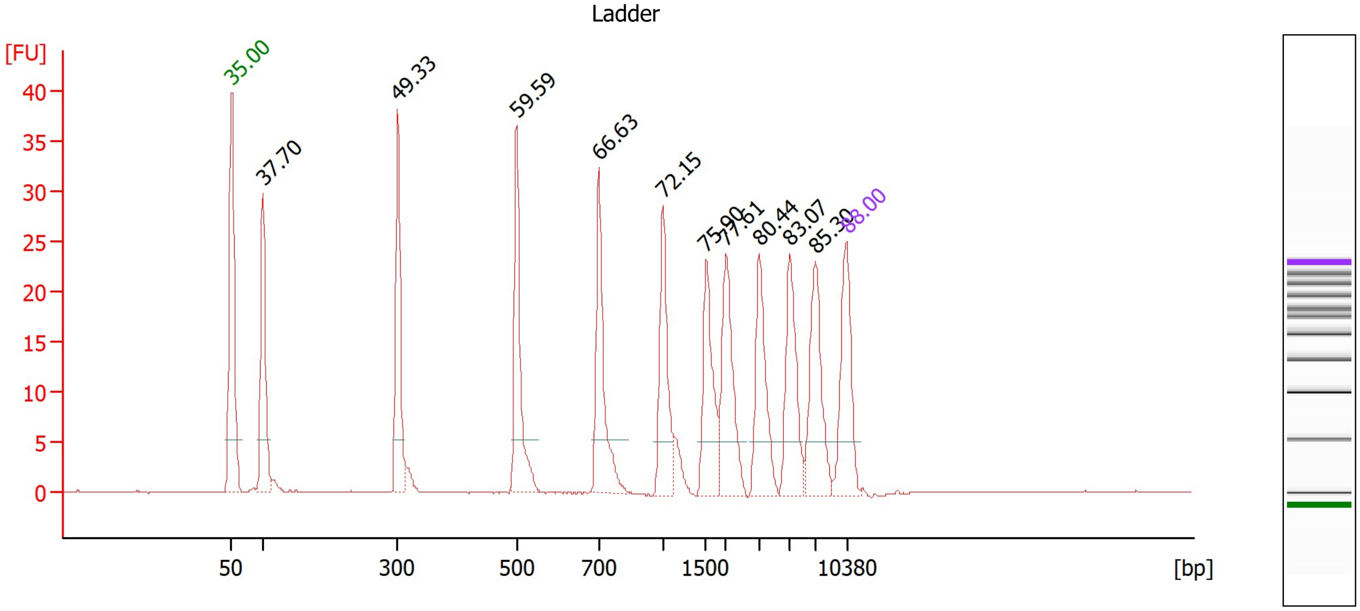

Peak table for Ladder

Peak		Size [bp]	Conc. [ng/µl]	Molarity [nmol/l]	Observations
1		50	8,30	251,5	Lower Marker
2		100	4,00	60,6	Ladder Peak
3		300	4,00	20,2	Ladder Peak
4		500	4,00	12,1	Ladder Peak
5		700	4,00	8,7	Ladder Peak
6		1 000	4,00	6,1	Ladder Peak
7		1 500	4,00	4,0	Ladder Peak
8		2 000	4,00	3,0	Ladder Peak
9		3 000	4,00	2,0	Ladder Peak
10		5 000	4,00	1,2	Ladder Peak
11		7 000	4,00	0,9	Ladder Peak
12		10 380	4,20	0,6	Upper Marker

 © Copyright 2003 - 2021 Agilent, Inc.

Assay Class: DNA 7500 Created: 2024-06-28 14:30:41
Data Path: C:\...-28\2100 expert_DNA 7500_DE72901282_2024-06-28_14-30-41.xad Modified: 2024-06-28 15:14:09

Electropherogram Summary Continued ...

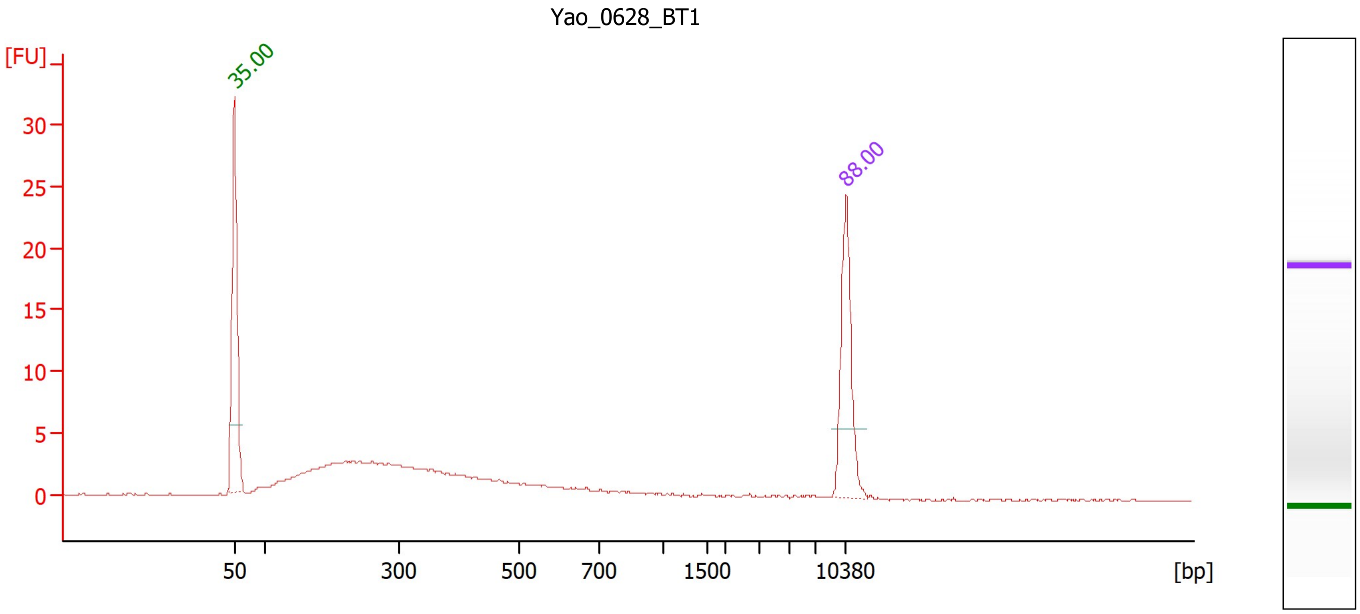

Overall Results for sample 1 : Yao_0628_BT1

Number of peaks found: 0

Peak table for sample 1 : Yao_0628_BT1

Peak		Size [bp]	Conc. [ng/µl]	Molarity [nmol/l]	Observations
1	◀	50	8,30	251,5	Lower Marker
2	▶	10 380	4,20	0,6	Upper Marker

 © Copyright 2003 - 2021 Agilent, Inc.

Assay Class: DNA 7500 Created: 2024-06-28 14:30:41
Data Path: C:\...-28\2100 expert_DNA 7500_DE72901282_2024-06-28_14-30-41.xad Modified: 2024-06-28 15:14:09

Electropherogram Summary Continued ...

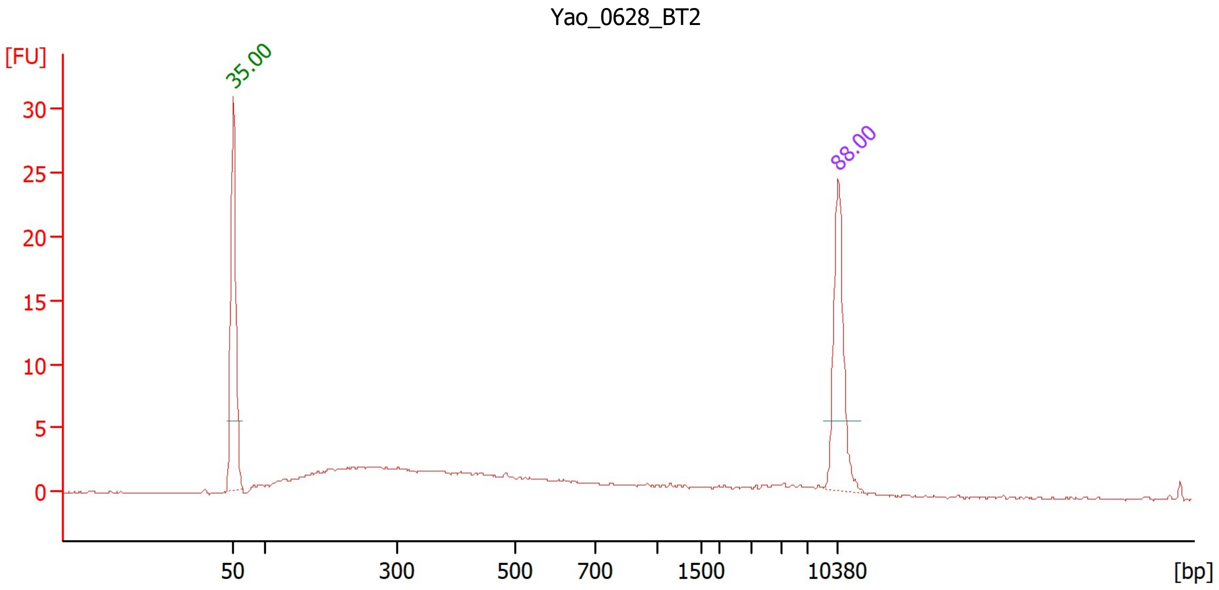

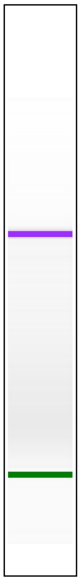

Overall Results for sample 2 : Yao_0628_BT2

Number of peaks found: 0

Peak table for sample 2 : Yao_0628_BT2

Peak		Size [bp]	Conc. [ng/µl]	Molarity [nmol/l]	Observations
1	◀	50	8,30	251,5	Lower Marker
2	▶	10 380	4,20	0,6	Upper Marker

 © Copyright 2003 - 2021 Agilent, Inc.

Assay Class: DNA 7500
Data Path: C:\...-28\2100 expert_DNA 7500_DE72901282_2024-06-28_14-30-41.xad
Created: 2024-06-28 14:30:41
Modified: 2024-06-28 15:14:09

Electropherogram Summary Continued ...

Yao_0628_BT3

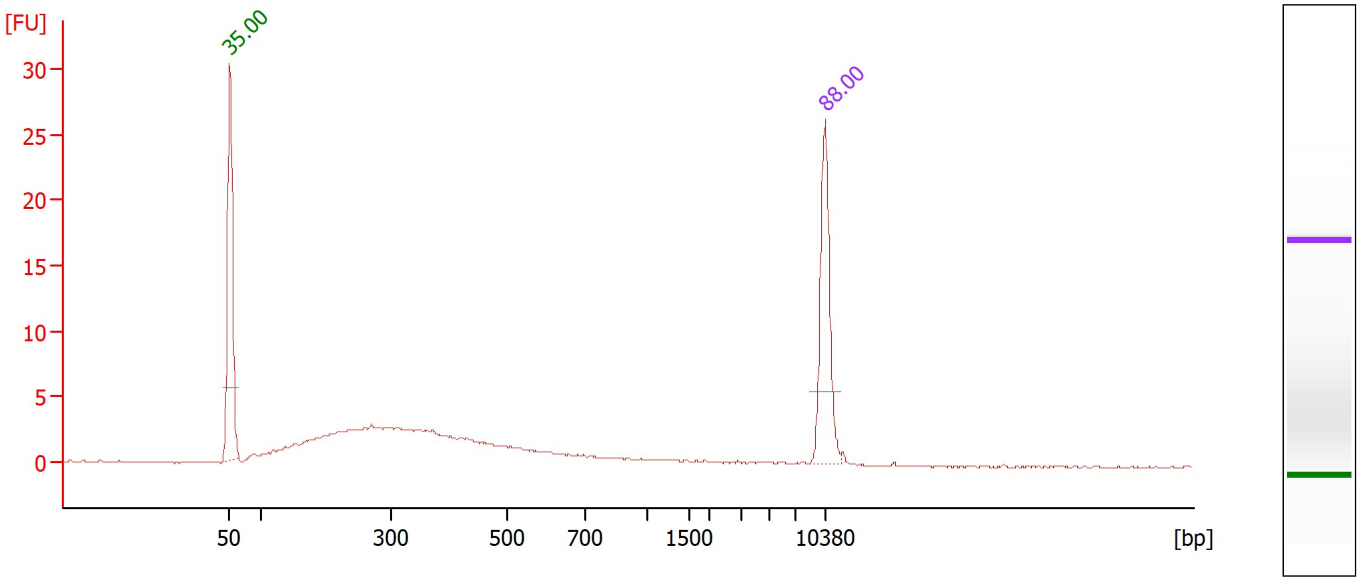

Overall Results for sample 3 : **Yao_0628_BT3**

Number of peaks found: 0

Peak table for sample 3 : **Yao_0628_BT3**

Peak		Size [bp]	Conc. [ng/µl]	Molarity [nmol/l]	Observations
1	◀	50	8,30	251,5	Lower Marker
2	▶	10 380	4,20	0,6	Upper Marker

© Copyright 2003 - 2021 Agilent, Inc.

Assay Class: DNA 7500 Created: 2024-06-28 14:30:41
Data Path: C:\...-28\2100 expert_DNA 7500_DE72901282_2024-06-28_14-30-41.xad Modified: 2024-06-28 15:14:09

Electropherogram Summary Continued ...

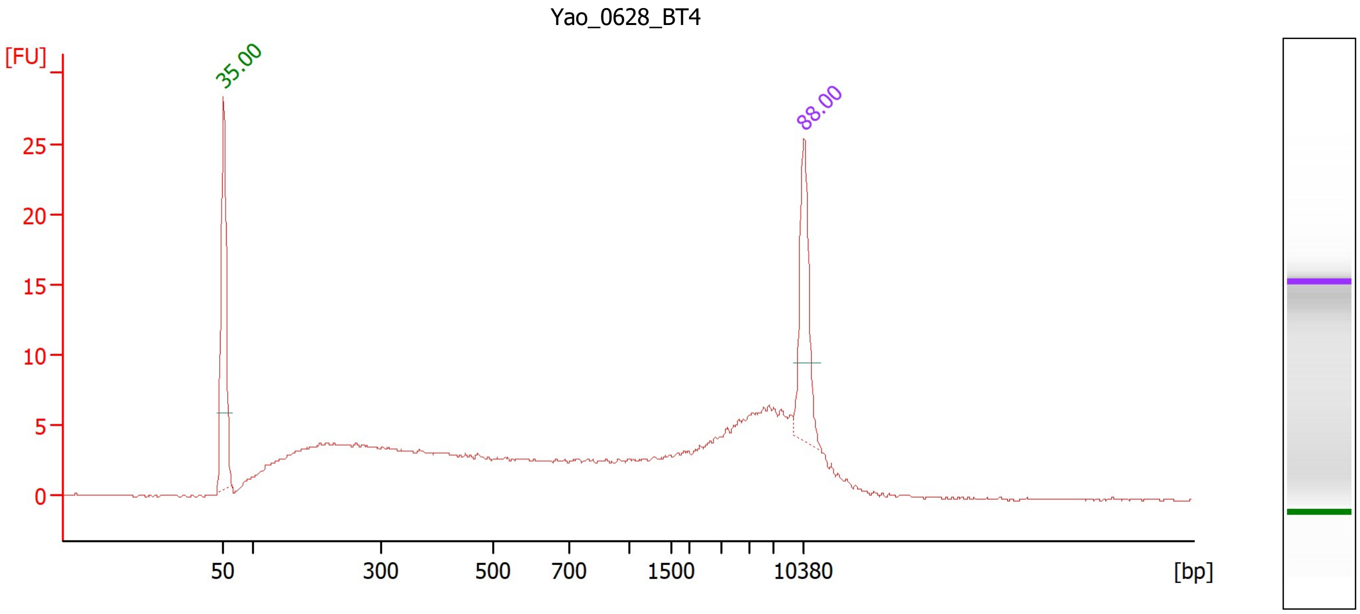

Overall Results for sample 4 : **Yao_0628_BT4**

Number of peaks found: 0

Peak table for sample 4 : **Yao_0628_BT4**

Peak		Size [bp]	Conc. [ng/µl]	Molarity [nmol/l]	Observations
1	◀	50	8,30	251,5	Lower Marker
2	▶	10 380	4,20	0,6	Upper Marker

 © Copyright 2003 - 2021 Agilent, Inc.

Assay Class: DNA 7500 Created: 2024-06-28 14:30:41
Data Path: C:\...-28\2100 expert_DNA 7500_DE72901282_2024-06-28_14-30-41.xad Modified: 2024-06-28 15:14:09

Electropherogram Summary Continued ...

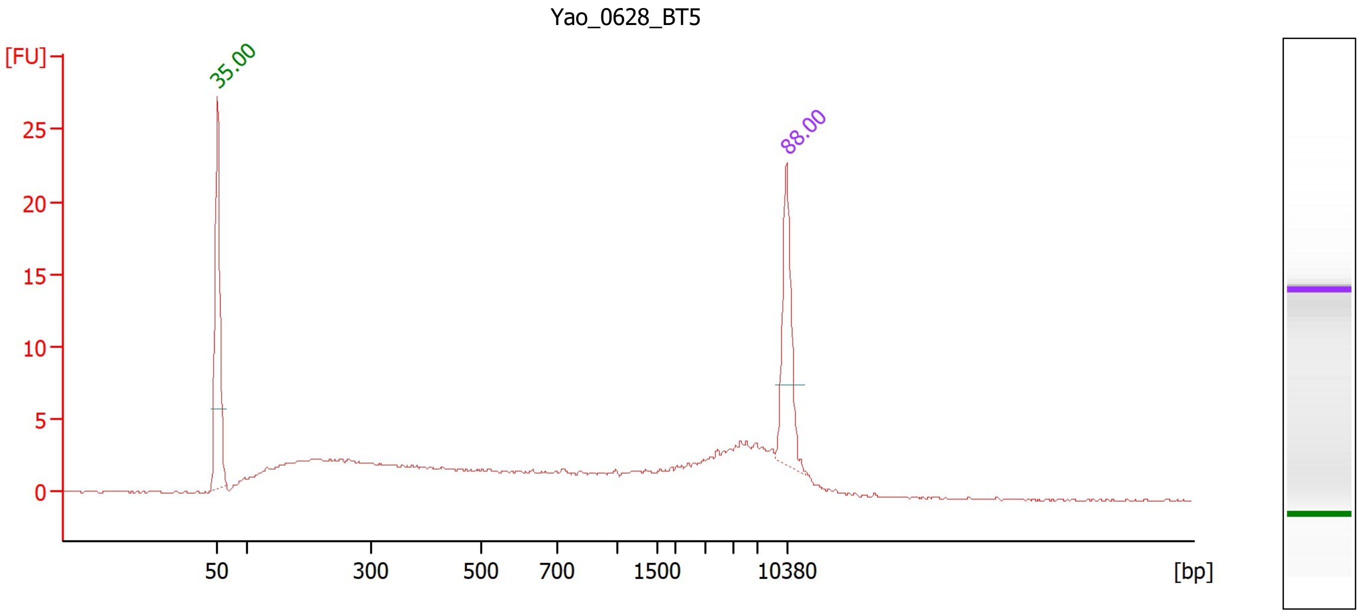

Overall Results for sample 5 : Yao_0628_BT5

Number of peaks found: 0

Peak table for sample 5 : Yao_0628_BT5

Peak		Size [bp]	Conc. [ng/µl]	Molarity [nmol/l]	Observations
1	◀	50	8,30	251,5	Lower Marker
2	▶	10 380	4,20	0,6	Upper Marker

 © Copyright 2003 - 2021 Agilent, Inc.

Assay Class: DNA 7500
Data Path: C:\...-28\2100 expert_DNA 7500_DE72901282_2024-06-28_14-30-41.xad
Created: 2024-06-28 14:30:41
Modified: 2024-06-28 15:14:09

Electropherogram Summary Continued ...

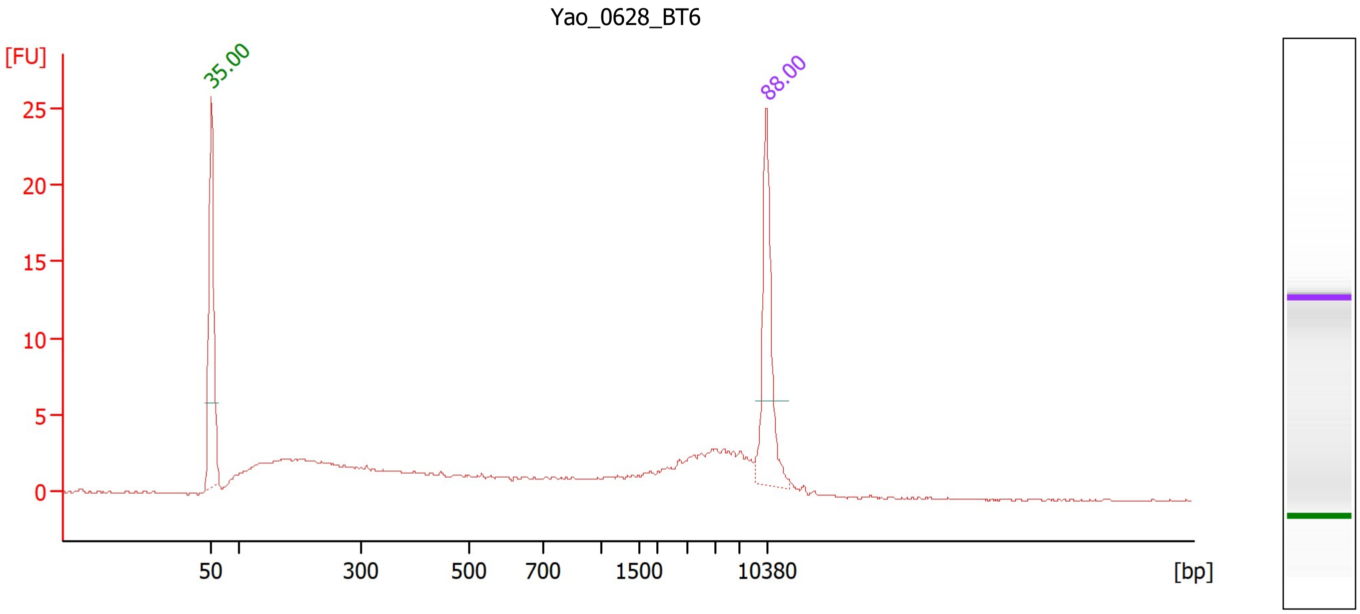

Overall Results for sample 6 : Yao_0628_BT6

Number of peaks found: 0

Peak table for sample 6 : Yao_0628_BT6

Peak		Size [bp]	Conc. [ng/µl]	Molarity [nmol/l]	Observations
1	◀	50	8,30	251,5	Lower Marker
2	▶	10 380	4,20	0,6	Upper Marker

 © Copyright 2003 - 2021 Agilent, Inc.

Assay Class: DNA 7500 Created: 2024-06-28 14:30:41
Data Path: C:\...-28\2100 expert_DNA 7500_DE72901282_2024-06-28_14-30-41.xad Modified: 2024-06-28 15:14:09

Electropherogram Summary Continued ...

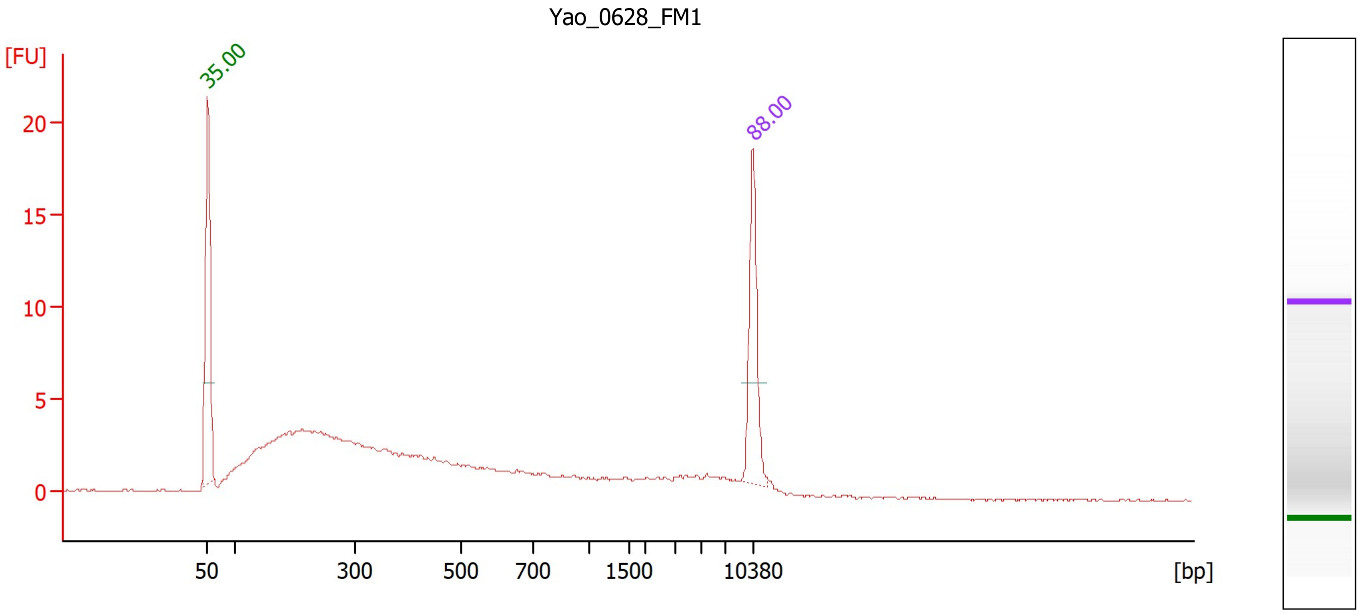

Overall Results for sample 7 : Yao_0628_FM1

Number of peaks found: 0

Peak table for sample 7 : Yao_0628_FM1

Peak		Size [bp]	Conc. [ng/µl]	Molarity [nmol/l]	Observations
1	◀	50	8,30	251,5	Lower Marker
2	▶	10 380	4,20	0,6	Upper Marker

 © Copyright 2003 - 2021 Agilent, Inc.

Assay Class:	DNA 7500	Created:	2024-06-28 14:30:41
Data Path:	C:\...-28\2100 expert_DNA 7500_DE72901282_2024-06-28_14-30-41.xad	Modified:	2024-06-28 15:14:09

Electropherogram Summary Continued ...

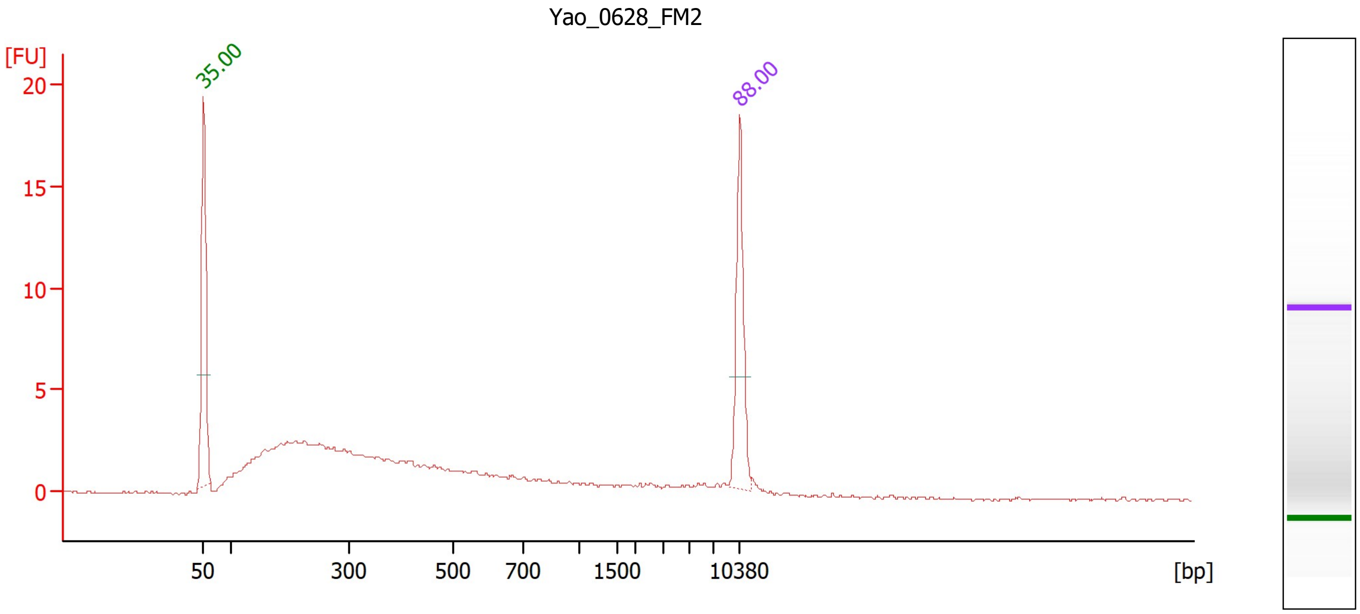

Overall Results for sample 8 : Yao_0628_FM2

Number of peaks found: 0

Peak table for sample 8 : Yao_0628_FM2

Peak		Size [bp]	Conc. [ng/µl]	Molarity [nmol/l]	Observations
1	◀	50	8,30	251,5	Lower Marker
2	▶	10 380	4,20	0,6	Upper Marker

 © Copyright 2003 - 2021 Agilent, Inc.

Assay Class: DNA 7500
Data Path: C:\...-28\2100 expert_DNA 7500_DE72901282_2024-06-28_14-30-41.xad
Created: 2024-06-28 14:30:41
Modified: 2024-06-28 15:14:09

Electropherogram Summary Continued ...

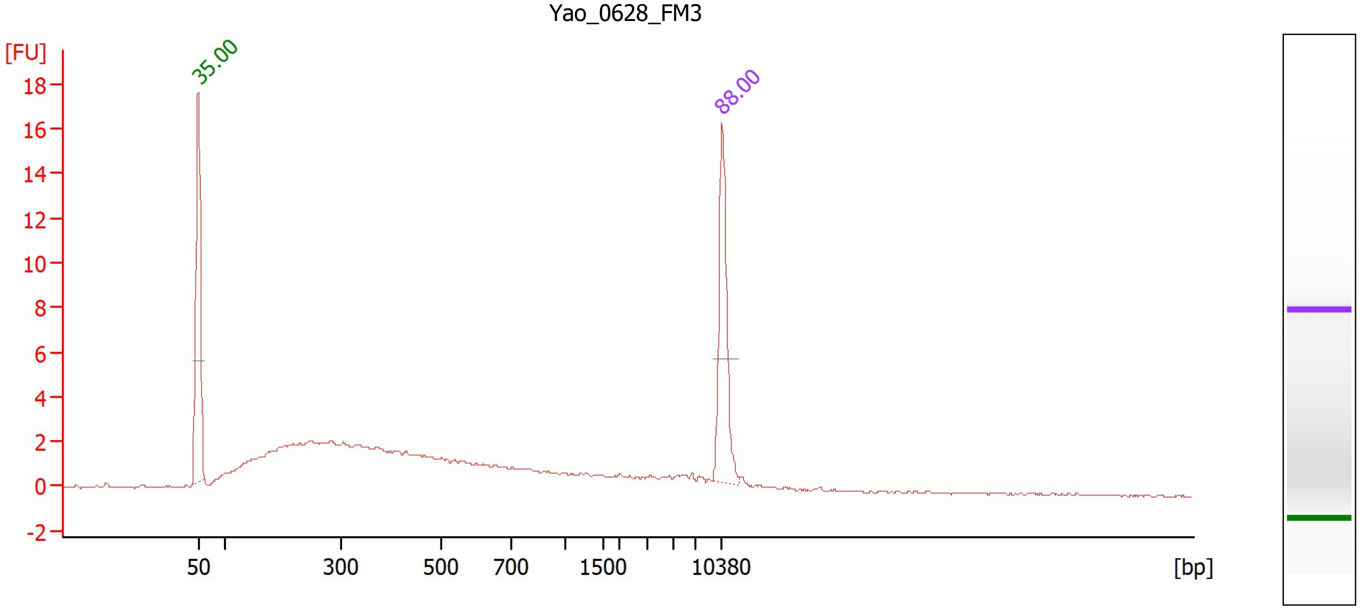

Overall Results for sample 9 : Yao_0628_FM3

Number of peaks found: 0

Peak table for sample 9 : Yao_0628_FM3

Peak		Size [bp]	Conc. [ng/µl]	Molarity [nmol/l]	Observations
1	◀	50	8,30	251,5	Lower Marker
2	▶	10 380	4,20	0,6	Upper Marker

© Copyright 2003 - 2021 Agilent, Inc.

Assay Class:	DNA 7500	Created:	2024-06-28 14:30:41
Data Path:	C:\...-28\2100 expert_DNA 7500_DE72901282_2024-06-28_14-30-41.xad	Modified:	2024-06-28 15:14:09

Electropherogram Summary Continued ...

Yao_0628_FM4

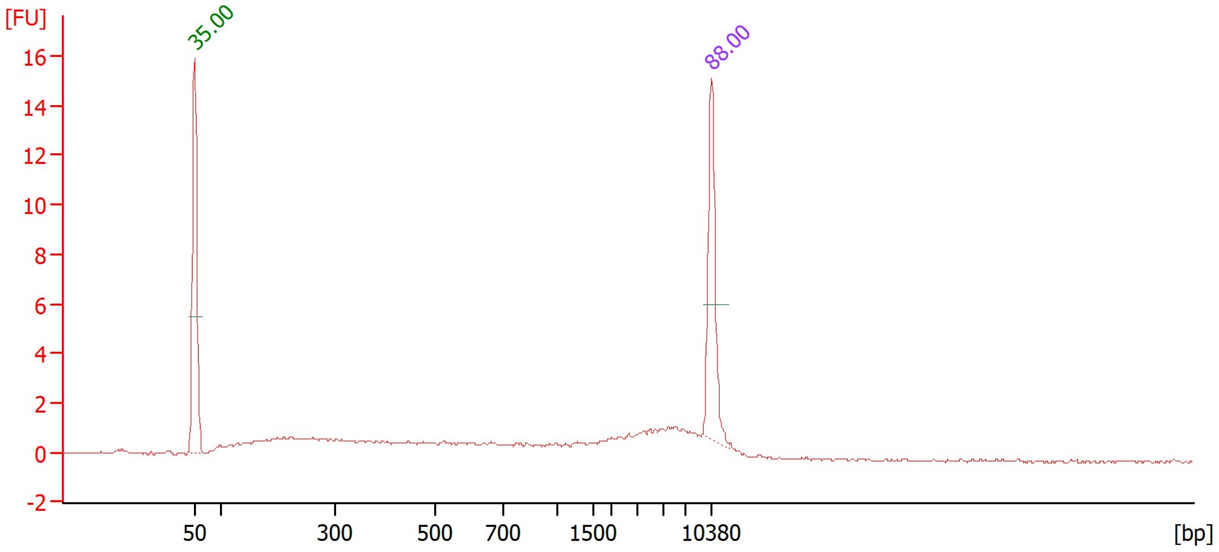

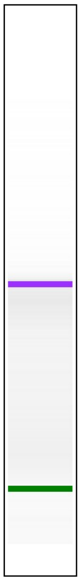

Overall Results for sample 10 : Yao_0628_FM4

Number of peaks found: 0

Peak table for sample 10 : Yao_0628_FM4

Peak		Size [bp]	Conc. [ng/µl]	Molarity [nmol/l]	Observations
1	◀	50	8,30	251,5	Lower Marker
2	▶	10 380	4,20	0,6	Upper Marker

 © Copyright 2003 - 2021 Agilent, Inc.

Assay Class: DNA 7500
Data Path: C:\...-28\2100 expert_DNA 7500_DE72901282_2024-06-28_14-30-41.xad
Created: 2024-06-28 14:30:41
Modified: 2024-06-28 15:14:09

Electropherogram Summary Continued ...

Yao_0628_FM5

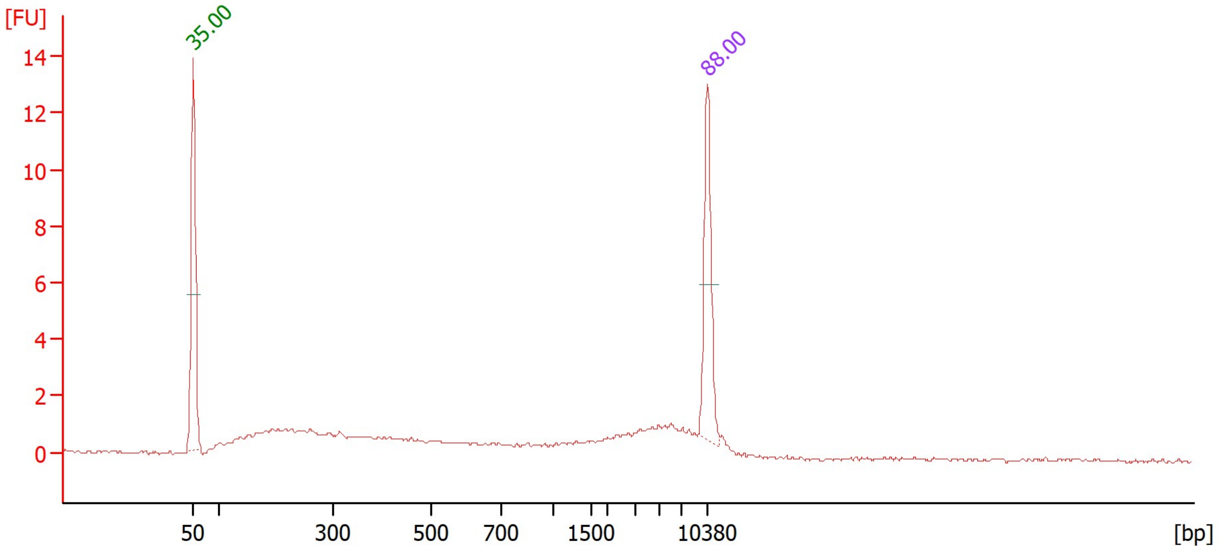

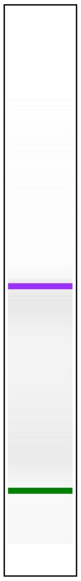

Overall Results for sample 11 : Yao_0628_FM5

Number of peaks found: 0

Peak table for sample 11 : Yao_0628_FM5

Peak		Size [bp]	Conc. [ng/µl]	Molarity [nmol/l]	Observations
1	◀	50	8,30	251,5	Lower Marker
2	▶	10 380	4,20	0,6	Upper Marker

 © Copyright 2003 - 2021 Agilent, Inc.

Assay Class: DNA 7500
Data Path: C:\...-28\2100 expert_DNA 7500_DE72901282_2024-06-28_14-30-41.xad

Created: 2024-06-28 14:30:41
Modified: 2024-06-28 15:14:09

Electropherogram Summary Continued ...

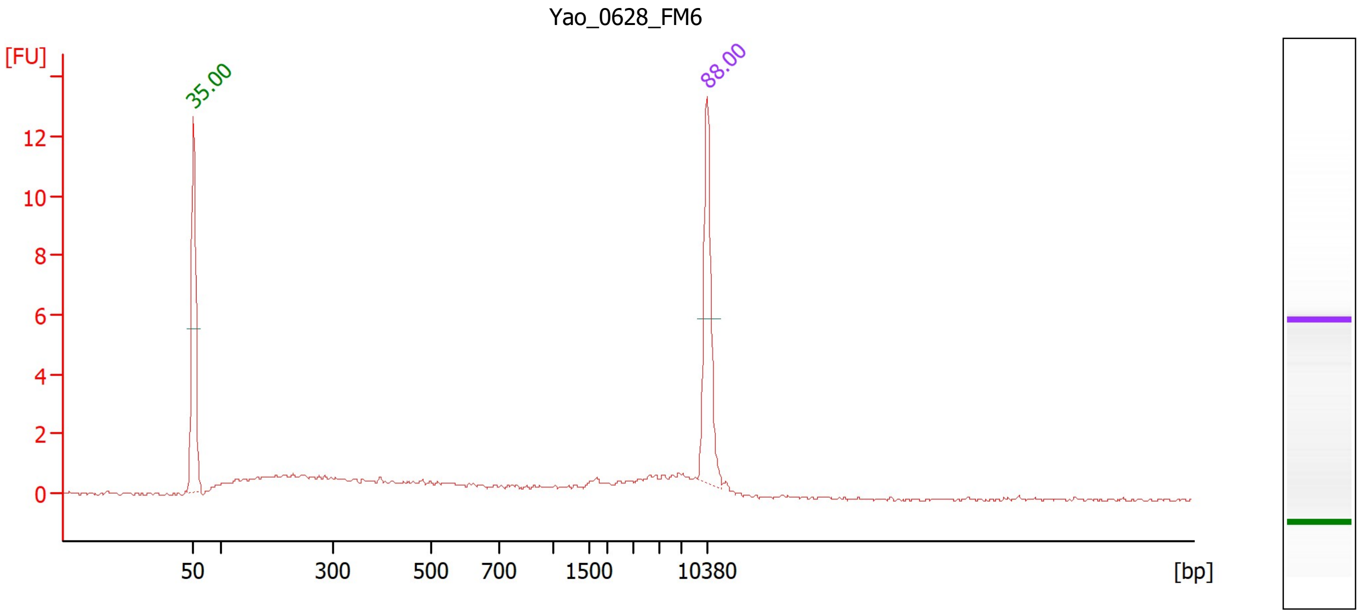

Overall Results for sample 12 : __Yao_0628_FM6__

Number of peaks found: 0

Peak table for sample 12 : __Yao_0628_FM6__

Peak		Size [bp]	Conc. [ng/µl]	Molarity [nmol/l]	Observations
1	◀	50	8,30	251,5	Lower Marker
2	▶	10 380	4,20	0,6	Upper Marker

 © Copyright 2003 - 2021 Agilent, Inc.

Assay Class: DNA 7500
Data Path: C:\...-28\2100 expert_DNA 7500_DE72901282_2024-06-28_14-30-41.xad
Created: 2024-06-28 14:30:41
Modified: 2024-06-28 15:14:09

Gel Image

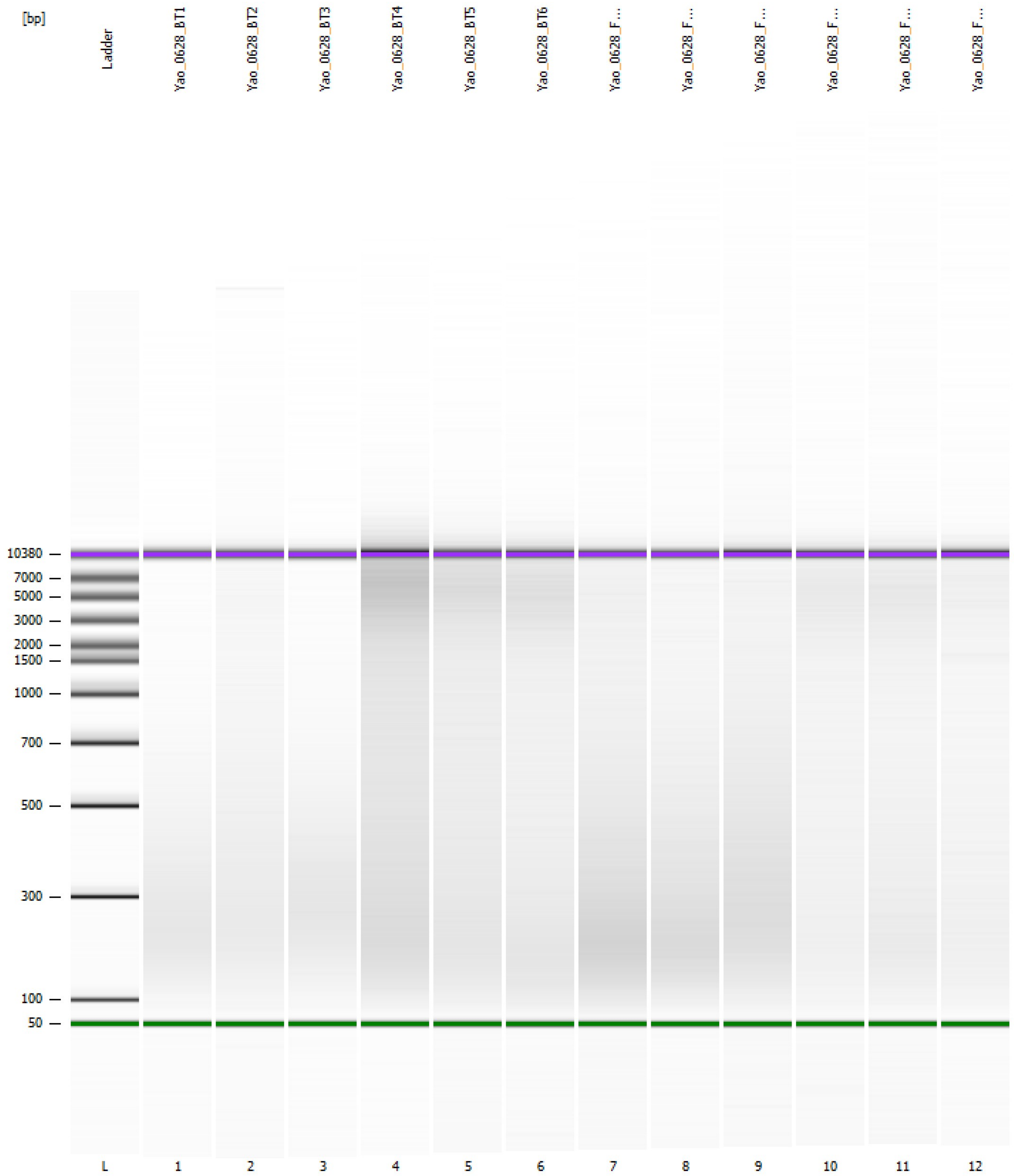

 © Copyright 2003 - 2021 Agilent, Inc.
